# Pigments, Chromatophore Structure, and Gene Expression Underlying Colour Polytypy of a Panamanian Poison Frog

**DOI:** 10.1111/mec.70214

**Published:** 2025-12-22

**Authors:** Vasiliki Mantzana‐Oikonomaki, Giselle Tamayo‐Castillo, Víctor Vásquez‐Cháves, Abel Mora‐Machado, William J. Zamora Ramirez, Roberto Ibáñez, Heike Pröhl, Ariel Rodríguez

**Affiliations:** ^1^ Institut für Zoologie, Stiftung Tierärztliche Hochschule Hannover Hannover Germany; ^2^ Centro de Investigaciones en Productos Naturales (CIPRONA) & Escuela de Química, Universidad de Costa Rica San Pedro Costa Rica; ^3^ CBio3 Laboratory, School of Chemistry University of Costa Rica San José Costa Rica; ^4^ Laboratory of Computational Toxicology and Artificial Intelligence (LaToxCIA), Biological Testing Laboratory (LEBi), University of Costa Rica San José Costa Rica; ^5^ Smithsonian Tropical Research Institute Panamá República de Panamá

**Keywords:** aposematic, cryptic, expression, genes, networks, pigmentation

## Abstract

Colour polytypism represents an example of phenotypic diversification shaped by genetic divergence and ecological pressures. Poison frogs of the genus *Oophaga* (Dendrobatidae) are highly polytypic in coloration, making them an ideal system for investigating the genetic and physiological basis of colour variation. We examined gene expression, pigment and histological differences across four colour morphs (aquamarine, brown, green, and red) of *Oophaga vicentei* from mainland Panama. RNA sequencing revealed 1838 and 5085 differentially expressed genes (DEGs) in the skin and liver, respectively. Melanin synthesis genes were upregulated in the brown morph, whereas pteridine pathway genes were upregulated in red and green morphs, consistent with previous findings in *O. pumilio* . In aquamarine frogs, pigment composition was similar to brown and green frogs, but transcriptional profiles were highly divergent. Red morphs upregulate a paralog of the dendrobatid ketolase *cyp3a80* in the liver, suggesting modified ketolation mechanisms in *O. vicentei* . This is consistent with higher ketocarotenoid accumulation in red frogs. Co‐expression network analysis identified morph‐related modules in both tissues but the relationship between modules and known pigmentation pathways remains unclear. Comparative analysis across seven dendrobatid species revealed conserved pigmentation genes (e.g., *xdh*, *ttc3b*) alongside morph‐specific expression patterns. Our results show that red frogs are dissimilar in pigments, chromatophore structure, and gene expression, whereas aquamarine, brown, and green coloration share overlapping pigment profiles and chromatophore organisation, with transcriptional differences suggesting structural or regulatory mechanisms.

## 1 Introduction

The presence of two or more distinct colour morphs within a natural population is referred to as colour polymorphism. Colour polytypism refers to colour diversity between populations of the same species resulting from genetic divergence at the population level (McLean and Stuart‐Fox 2014). Such divergence may be the result of stochastic evolutionary processes, like genetic drift (Roulin 2004; Svensson 2017), or of adaptive differentiation driven by selection. Coloration may serve ecological, behavioural or physiological functions such as thermoregulation (Thompson et al. 2023), mate attraction (Wellenreuther et al. 2014) and anti‐predator strategies (Endler and Mappes 2017). Aposematism is a widespread predation avoidance strategy where prey species use bright, conspicuous coloration to signal unpalatability or other defensive traits (Ruxton et al. 2018). Population diversity in colour signals typically emerge because of spatially differential predation pressures leading to colour variation between populations (Briolat et al. 2019; Nokelainen et al. 2024). Clarifying the genetic and molecular basis for this colour variation is central to our comprehension of the evolutionary pressures creating and maintaining such striking colours.

Coloration in vertebrates, such as fish, reptiles, and amphibians, is produced by chromatophores. Chromatophores are cells that either contain pigments, the compounds responsible for different colours, or nanostructures reflecting light that result in structural coloration (Sköld et al. 2012; Ligon and McCartney 2016). Structure and composition of chromatophore cells are interacting and contribute to the final colouration (Bagnara et al. 1968; Shawkey and D'Alba 2017). Three types of chromatophores are xanthophores, iridophores and melanophores that form a unit known as the chromatophore unit (Bagnara and Hadley 1973). Xanthophores predominantly form the outermost layer and possess pteridines and carotenoids that impart yellow and red colouration. Underneath the xanthophores are the iridophore cells, containing guanine crystals and reflecting light to impart iridescent or structural colouration (Bagnara and Hadley 1973). Finally, the melanophores contain melanin and contribute black, brown, or dark pigmentation which can deepen or complement the colour contributed by the overlying layers (Bagnara and Hadley 1973). The layers interact, and pigment distribution within them plays a vital role in deepening or changing the animal's skin colour.

Recent studies have identified several genes that are responsible for variations in skin colour among vertebrate lineages. In mammals, for instance, domestic cat coat colour variation is melanophore‐based and colour variation is influenced by several genes, including genes for tyrosinase family enzymes (e.g., *tyr*, *tyrp1*; see Table 1 for gene name from abbreviation) all important for the melanin synthesis pathway. Deficiency of the *tyr* gene is responsible for the albino cat and specific mutations to Siamese and Burmese phenotypes (pigmented ears, tail, and paws) (Schmidt‐Küntzel et al. 2005). While melanophore‐mediated pigmentation is well characterised in mammals due to its relevance to human pigmentation, much less is known in other vertebrates regarding the various mechanisms that lead to colour production.

**TABLE 1:** Gene abbreviations and full names. Abbreviations listed here are used throughout the manuscript to refer to their corresponding full gene names.

Abbreviation	Full gene name
*tyr*	Tyrosinase
*tyrp1*	Tyrosinase‐related protein 1
*mc1r*	melanocortin 1 receptor gene
*pmel*	premelanosome protein
*spr*	Sepiapterin reductase
*bco2*	Beta‐carotene oxygenase 2
*mitf*	Melanocyte inducing transcription factor
*slc24a5*	Solute Carrier Family 24 Member 5
*oca2*	Oculocutaneous albinism II
*hells*	Helicase, lymphoid specific
*xdh*	Xanthine dehydrogenase
*ctsd*	Cathepsin D
*dgat2*	Diacylglycerol O‐acyltransferase 2
*pomc*	Proopiomelanocortin
*α‐MSH*	Alpha‐melanocyte stimulating hormone
*tcf7*	Transcription factor 7
*wnt16*	Wnt Family Member 16
*tyms*	Thymidylate synthetase
*ttc39b*	Tetratricopeptide repeat domain 39B
*cyp2j19*	Cytochrome P450 Family 2 Subfamily J Member 19
*cyp2g1*	Cytochrome P450 Family 2 Subfamily G Member 1
*cyp3a80*	Cytochrome P450 Family 3 Subfamily A Member 80
*rbp2*	Retinol binding protein 2
*cyp26b1*	Cytochrome P450 Family 26 Subfamily B Member 1
*cyp3a82*	Cytochrome P450 Family 3 subfamily A Member 82
*cyp27a1*	Sterol 27‐hydroxylase
*npc1l1*	NPC1‐like intracellular cholesterol transporter 1
*aldh1a2*	Aldehyde Dehydrogenase 1 Family Member A2
*r*ara	Retinoic Acid Receptor Alpha
*stard3*	StAR Related Lipid Transfer Domain Containing 3
*liph*	Lipase H (Triacylglycerol Lipase)
*rpia*	Ribose‐5‐phosphate isomerase A
*usp43*	Ubiquitin specific peptidase 43

However, recent research has shown a growing interest toward non‐model organisms and has revealed a greater variety of genetic and cellular mechanisms that underlie coloration. For example, in the common wall lizard, *Podarcis muralis* , colour variation between morphs is associated with gene activity in the pteridine pathway (e.g., *spr*) and carotenoid metabolism (e.g., *bco2*; Andrade et al. 2019). In cichlid fish, *mitf*, *slc24a5*, and *oca2* genes regulate pigment cell development and melanosome formation to support diverse coloration between species (Wang et al. 2021). In the pumpkin toadlet *Brachycephalus actaeus* (Monteiro et al. 2018), patterns of different melanin‐related gene expression (e.g., *tyrp1*) were associated with different colour morphs and patterns of chromatophore organisation (Monteiro et al. 2024). These findings highlight the complex regulatory mechanisms involved in skin pigmentation of polymorphic species.

The Dendrobatidae, a family of Neotropical poison frogs, are renowned for the diversity of vibrant colour morphs, which are closely associated with chemical defences (Summers and Clough 2001; Bolton et al. 2017). In some lineages there are morphs that use warning coloration to advertise toxicity and avoid predators, while other morphs are cryptic and utilise background matching for predator avoidance (Saporito et al. 2007; McElroy 2016). Differential gene expression studies across colour morphs of *Ranitomeya*, *Dendrobates* and *Oophaga* species have identified multiple colour related genes involved in melanin (e.g., *tyr, tyrp1, mc1r, pmel*), pteridine (*xdh*) and carotenoid pathways (*bco2, rbp1, dgat2*). Many of these genes are differentially expressed between morphs in *D. auratus* , *R. imitator* , *R. variabilis* and *O. sylvatica* , highlighting the polygenic nature of coloration in Dendrobatids (Posso‐Terranova and Andrés 2020; Stuckert et al. 2019, 2023; Rubio et al. 2024). Recently, transcriptome analysis of the red and yellow morphs of *Ranitomeya sirensis* revealed that a gene in the cytochrome P450 family, *cyp3a80*, could be an amphibian‐specific ketolase converting β‐carotenes into red ketocarotenoids in the livers of the red frogs (Twomey, Johnson, et al. 2020; Twomey, Kain, et al. 2020).

One of the most studied species for aposematism is the Strawberry Poison Frog ( *Oophaga pumilio* ; Schmidt 1857). Across the Bocas del Toro archipelago in Panama, *Oophaga pumilio* populations display exaggerated colour variation, varying from camouflaging brown and green to bright red and orange morphs, with the more conspicuous morphs being more toxic (Summers and Clough 2001; Maan and Cummings 2012). Geographical isolation on the islands, accompanied by genetic drift, sexual selection and predation pressure, have been suggested as key factors promoting colour polytypism (Rudh et al. 2007; Wang and Summers 2010; Dreher et al. 2015; Gade et al. 2016). Differential gene expression analyses between red, green and blue colour morphs of *O. pumilio* have shown differences in the expression of genes related to melanin synthesis and carotenoid metabolism, like *bco2*, *slc24a5* and *tyrp1* (Rodríguez et al. 2020). The gene *ttc39b* was associated with the evolutionary shift from red to yellow coloration in *O. pumilio* , highlighting the importance of colour gene regulation for colour diversification (Aguilar‐Gómez et al. 2024).

*Oophaga vicentei* (Jungfer et al. 1996) is a little‐known and endangered species (listed as Endangered under criteria B1ab on the IUCN Red List, IUCN SSC Amphibian Specialist Group 2019), native to the Caribbean versant of mainland Panama. Like its closest relative *O. pumilio* , *O. vicentei* exhibits remarkable polytypism, with morphs ranging from bright red to dark brown (Lötters et al. 2008; Monteiro et al. 2023). Unlike its close relative *O. pumilio* , whose colour diversification is mostly associated with geographic barriers, *O. vicentei* displays extensive colour polytypism across the Panamanian mainland. This makes it an ideal system to investigate the mechanism by which such striking colour divergence can evolve and be maintained in the absence of geographic barriers. In this study, we characterise the variation in dorsal skin spectral reflectance, colour pattern, histology, and pigment composition in individuals from four colour morphs of *O. vicentei* and relate this variation to the gene expression in skin and liver. We hypothesise that the abundance of specific carotenoids and pteridines as well as some histological characteristics of the skin will be associated with the dorsal colour differentiation observed in this species and that the gene expression differences between the colour morphs in the skin and liver will provide insights into the gene regulatory processes and key molecular mechanisms responsible for the striking polytypism in this species.

## 2 Methods

### 2.1 Sampling

Fieldwork was conducted in the provinces of Colón and Veraguas, Panama, during 2022 and 2023. In total, we sampled 21 male individuals from four distinct localities with different colour morphs: La Ceiba, Colón (CEI, 8.808, −80.607; *N* = 8), Empalizada (EMP, 8.72, −81.192; *N* = 5), Loma Grande (LOM, 8.541, −81.157; *N* = 4), and Calovebora (CAL, 8.789, −81.214; *N* = 4). Following capture, frogs were placed in individual bags with leaf litter to maintain moisture and were transported to the field station for sample processing within 1–2 h. Sexual dimorphism in colour is absent in *Oophaga* (Lötters et al. 2008) and since vocalising males are easier to find and females might be the limiting sex in reproduction as in *Oophaga pumilio* (Pröhl and Hödl 1999), only males were sampled.

### 2.2 Spectrometry

We measured dorsal skin spectral reflectance (R) of captured male frogs using an R‐200‐2‐UV/Vis fibre optic probe connected to an HR2000 spectrometer with a deuterium‐tungsten DT‐Mini‐2‐GS lamp (Ocean Optics Inc., USA). The spectrometer was calibrated with a WS‐1‐SS white standard before each measurement. Reflectance spectra were exported via OceanView 2.0.10 software (Ocean Optics Inc., USA). Measurements were taken in a dark room to minimise ambient light. The fibre optic probe was held 2 mm above the dorsal surface using a dark cylindrical adapter, and illumination was provided by the deuterium‐tungsten DT‐Mini‐2GS lamp attached to the spectrometer. Four points were measured per frog in an anterior–posterior zig‐zag pattern (i.e., anterior‐left, anterior‐right, posterior‐left, posterior‐right) to capture variation across the dorsal surface. Spectra were processed in R using the pavo package (Maia et al. 2019; R Core Team 2024). Spectra were trimmed to 300–700 nm, smoothed (0.2 coefficient), and negative values were set to zero. Spectra were averaged per individual, and six spectral shape descriptors mean brightness (B2) and chroma in the violet, blue, green, yellow, and red wavelengths (S1V, S1B, S1G, S1Y, S1R) were extracted using the summary function in pavo.

### 2.3 Colour Pattern Analysis

We captured standardised UV and visual range images using a modified Samsung NX1000 mirrorless camera with a Novoflex Noflexar 35 mm lens. For visual range images we used a Baader UV/IR cut filter, and for UV images a Baader U Filter (Baader Planetarium, GmbH, Germany). The setup followed standardised biological photography guidelines (Empirical Imaging 2025). The setup included consistent camera positioning, settings (ISO, aperture, shutter speed), and a spectrally neutral background (grey). Imaging was done in a dark room, and an Iwasaki eyeCOLOUR lamp (CP‐lighting, UK) without UV filter was used as light source. For lighting diffusion, a natural white PTFE was positioned around the photographed subject. Each image included a grey‐scale calibration standard for accurate reflectance calibration and was saved in RAW format.

Images were processed in Micatoolbox v2.2 (Troscianko and Stevens 2015) using the Quantitative Colour and Pattern Analysis (QCPA) framework (van den Berg et al. 2020) with the blue tit as a visual model, which represents a conserved, tetrachromatic vision of diurnal birds. Birds are widely considered among the main predators of *Oophaga* frogs (Master 1999; Poulin et al. 2001; Saporito et al. 2007; Santos and Cannatella 2011; Willink et al. 2014; Dreher et al. 2015; Paluh et al. 2015). Gaussian spatial acuity correction and RNL clustering were enabled, using receptor noise‐limited models with a Weber fraction of 0.05 (photoreceptors) and 0.1 (luminance). Aquity value was set at 6.6 cycles per degree, based on data from forest passerines (Troscianko and Stevens 2015; Moore et al. 2013) and distance or value at 1.5 m distance (van den Berg et al. 2020). From the output of the QCPA (van den Berg et al. 2020), we selected 10 non‐redundant colour pattern variables resulting from the colour adjacency, visual contrast and boundary strength analyses (CAA_Jc: relative Simpson colour diversity, CAA_Jt: relative Simpson transition diversity, CAA_C: pattern complexity, CAA_PT: average patch size, CAA_Asp: patch aspect ratio, VCA_MS: weighted mean of pattern RNL chromaticity contrast, VCA_MSL: weighted mean of RNL luminance pattern contrast, BSA_BML: weighted mean of luminance boundary strength, BSA_BMDmax: weighted mean of chromaticity boundary strength, and BSA_BMS: weighted mean of RNL chromaticity boundary strength).

### 2.4 Tissue Collection

After collecting spectral measurements and multispectral images, each frog was immersed in MS222 until anaesthesia and euthanised by cerebral pithing. Specimens were then quickly dissected to collect the dorsal skin and liver. Half of each tissue was preserved in 2 mL of RNAprotect (QIAGEN, Hilden, Germany) solution before being stored at −80°C for future RNA extraction. The other half was preserved in 2.0 mL SureSTAR (ThermoFisher, USA) vial prefilled with 1.5 mL methanol and transported under cold conditions to the lab. A small section of the dorsal skin, located between the head and the trunk, was also collected and preserved in formaldehyde for histological preparation. This section was the same colour as the rest of the dorsal area.

### 2.5 Histology and Image Analysis

Formaldehyde‐fixed skin samples were used to prepare histological slides for bright‐field microscopy (BFM). After a second fixation, samples were treated with osmium tetroxide, dehydrated in ethanol, and embedded in Epon 812 resin. Semithin (1–10 μm) sections were cut with an ultramicrotome, stained with toluidine blue, and imaged using a ZEISS Axioplan 2 microscope at 100×, 200×, and 400× magnification. Chromatophore presence and distribution were analysed in ImageJ (ImageJ 1.51q; Schneider et al. 2012). Layer thickness was measured from images of 400× magnification captured with a DFK 23UX249 camera using IC Capture 2.4 software. Colour adjustments were standardised (Brightness: 465; Contrast: 13; Auto ref.: 155), and six images per sample were taken at 1920 × 1200 resolution calibrated using a Thoma‐type hemacytometer with a 2.5 μm scale to enable accurate scaling of image measurements. This calibration allowed us to obtain representative physical measurements of chromatophore layer thickness for each cell type. Thickness values were then scaled to micrometres (μm) and averaged per cell type and individual. Separate ANOVA tests were performed to estimate the effect of morph on xanthophore, melanophore, and iridophore mean layer thickness, after log transformation of measurements to fit the normality assumption requirements. Iridophore platelet orientation is known to influence hue (Twomey, Kain, et al. 2020), but due to degradation in several samples, our histological analysis focused on quantifying chromatophore layer thickness.

### 2.6 Carotenoid and Pterins Profiling

Tissue samples were subjected to repeated freeze–thaw cycles (−70°C and room temperature), then sonicated (BRANSON 3800, 15 min) and centrifuged (Thermo Scientific, 4500 rpm, 10 min). The supernatant was collected for analysis and residual tissues were dried and weighed. For skin extraction preparations, an aliquot of 90 μL of extract was supplemented with 10 μL of nicotine (Sigma‐Aldrich, 0.1 μg/μL) as an internal standard, in a 250 μL SureSTART glass insert and then submitted to UPLC‐HRMS. Separation of pigments was obtained using a Vanquish Ultra Efficient Liquid Chromatograph equipped with a photodiode array detector, an Acquity Premier BEH C18 column (1.7 mm, 2.1 × 100 mm) and using as solvent A, a mixture of methanol: acetonitrile 3:7 V/V, and solvent B, water with 0.01% (V/V) of formic acid, as described in Data S1. Calibration curves using astaxanthin and β‐carotene were used. High resolution mass spectra were obtained using an Orbitrap Exploris 120 mass spectrometer (Thermo Scientific). Detection succeeded with ESI positive ionisation for oxygenated carotenoids and APCI positive ionisation for lipophilic carotenoids as described in Data S1. Details on mass spectrometry, MS^1^ (full scan) and MS^2^ (tandem MS) data processing, including calibration curves and compound annotation workflows, are provided in Data S1.

QCPA, spectral, histological, and carotenoid‐related variables were combined to capture the visual and cellular basis of coloration. A principal component analysis (PCA) was performed to reduce dimensionality and identify which variables contributed most to the variation among morphs. Variables with the highest loadings on the first principal components were selected for downstream analysis, as these represent the traits most responsible for differences in visual appearance and pigmentation.

### 2.7 RNA Extraction and High‐Throughput Sequencing

RNA was extracted from all tissue samples using the QIAwave RNA Mini Kit (QIAGEN, Hilden, Germany) and QIAshredder (QIAGEN), following the manufacturer's protocol with following modifications: disruption with a stainless‐steel pestle followed by homogenization on QIAshredder columns (QIAGEN, Hilden, Germany) and an added centrifugation step at 14,000 rpm for 3 min. RNA concentration was quantified with the Qubit RNA High Sensitivity Assay Kit (Thermo Fisher Scientific Inc., Germany) with concentrations ranging from 100 to 500 ng/μl.

Library preparation and strand‐specific RNA sequencing with polyA selection were performed by Genewiz (Leipzig, Germany) using the Illumina NovaSeq platform with 2 × 150 bp reads. The raw sequencing data are publicly available on NCBI SRA bioproject PRJNA1234489.

### 2.8 Transcriptome Assembly

Before assembly, read quality was assessed with FASTQC v0.12.0 (Andrews 2010), and FastP 0.23.4 (Chen et al. 2018) was used for adapter removal and trimming. Contaminants were screened with FastQ Screen v0.15.1 (Wingett and Andrews 2018) and Bowtie2 v 2.5.1 (Langmead and Salzberg 2012), while Kraken2 v2.1.3 (Wood et al. 2019) was used to remove potential microbiome or sampling contaminants. Sequencing errors were corrected with Rcorrector v3 (Song and Florea 2015). Cleaned reads were concatenated by locality to reconstruct site‐specific transcriptomes, reducing locality‐related genetic noise. Reads were normalised with BBnorm (bbtools v39.08; Bushnell 2014), and rRNA was removed using SortMeRNA v4.3.6 (Kopylova et al. 2012).

No reference genome exists for *O. vicentei* , and to select the best reference to estimate the gene expression in the target species we compared three alternative approaches: (1) assemble the transcriptome of *O. vicentei* denovo, (2) conduct a genome‐guided assembly of *O. vicentei* transcriptome using the genome of *O. pumilio* , and (3) same but using the genome of *O. sylvatica* as reference. The comparison of these three assemblies in terms of mapping rate, contiguity and completeness clearly indicated that the de novo approach was superior in our case (Table A1).

De novo transcriptomes were first assembled at locality level using five different assemblers: Trinity v2.15.1 (Grabherr et al. 2011), SOAP‐denovo‐trans v1.0.5 (Xie et al. 2014), rnaSPADES v3.15.4 (Bushmanova et al. 2019), idba‐trans v1.1.3 (Peng et al. 2013), and Velveth‐OASES v3 (Schulz et al. 2012). We generated one de novo transcriptome per locality with Trinity using rRNA‐filtered, paired‐end reads in RF orientation with a minimum k‐mer coverage of 3. SOAPdenovo‐Trans optimised contiguity with multiple k‐mer values (19, 37, 55, 73), producing four assemblies per locality (16 total), using a read length of 150 bp and an average insert size of 230 bp, verified with FastP. rnaSPADES assembled one transcriptome per locality using automatic k‐mer selection and strand‐specific RF mode. IDBA‐Trans also produced one assembly per locality, converting FASTQ to FASTA while merging reads, then assembling across a k‐mer range of 19–73 with a maximum of 50 isoforms. Velvet‐OASES used the same four k‐mers (19, 37, 55, 73), processing paired‐end reads separately via Velvet for contigs and Oases for isoform merging, resulting in four assemblies per locality. In total, this approach generated 14 transcriptome assemblies per locality.

The multiple resulting transcriptome assemblies were integrated into a comprehensive reference set using EvidentialGene pipeline vJUL‐2023 (Gilbert 2013). EvidentialGene retains biologically relevant isoforms by prioritising the longest and most complete coding sequences (CDs) while preserving alternative isoforms that meet quality thresholds of a minimum protein length of 70 amino acids and a heterozygosity threshold of 2%. During de‐duplication, redundant fragments were removed, but distinct isoforms were maintained in the final transcriptome and included in downstream expression analyses. The final transcriptome represents a non‐redundant transcriptome. The completeness and quality of the resulting transcriptome were assessed with BUSCO v5.7.1 (Simão et al. 2015; Manni et al. 2021) against the vertebrate odb10 database and rnaQUAST v2.3.1 (Bushmanova et al. 2016). To annotate the assembled transcripts, the EnTAP v1.3.3 pipeline (Hart et al. 2020) was configured with UniProt's SwissProt, TrEMBL [Amphibia subset], RefSeq [vertebrate subset], and UniRef90 databases (The UniProt Consortium 2023; O'Leary et al. 2016). A 0.5 FPKM cutoff was used to filter out low‐expressed transcripts and a minimum protein length of 100 amino acids was set on the frame selection step. Annotated contaminants from human, mouse, fungi, nematodes, bacteria, viruses, and plants were filtered out. Amphibia was favoured taxonomically, and ontology analysis was conducted using EggNOG (Huerta‐Cepas et al. 2019). To obtain gene‐level expression estimates in subsequent analyses, the transcript to gene map result from EvidencialGene was used to collate all transcript isoforms into genes. Gene descriptions and symbols were assigned to the genes based on BLAST results, prioritising matches to *Xenopus tropicalis* orthologs (reciprocal best blast hits on NCBI RefSeq assembly GCF_000004195.4, e‐value: 1e‐5), followed by EnTAP hits and finally EggNOG gene family names, if no closer match was found. When multiple *O. vicentei* transcripts matched the same gene name, these were ranked in decreasing bit‐scores and the ranks were added as suffixes to the gene name to all but the top scoring transcript to indicate potential paralogs.

### 2.9 Differential Gene Expression

We identified differentially expressed (DE) genes among morphs using the limma‐voom method (v3.60.6; Law et al. 2014), chosen for its flexibility in handling multiple comparisons. We estimated transcript abundances by pseudoaligning the quality trimmed reads onto the reference transcriptome using Kallisto v0.50.1 (Bray et al. 2016) with 100 bootstrap pseudo‐replicates. These estimates were then used in the limma‐voom DE analysis, conducted separately for skin and liver tissues. Expression estimates were then used in the limma‐voom DE analysis, which was conducted separately for skin and liver (see 3. Results).

Expression estimates per transcript were collated into gene‐level estimates using a transcript‐to‐gene mapping table result from the EvidentialGene pipeline, filtered to include only transcripts with open reading frames (ORFs) and annotations. Filtering of genes with low expression was done using the filterByExpr function of edgeR (v4.2.2; Chen et al. 2014) with the filtering guided by the experimental design to account for morph‐specific expression patterns. The model design included the variable “morph” as an explanatory factor. Specifically, a gene was retained if it had counts above 5 in at least the minimum number of 2 samples (i.e., accounting for morph‐specific expression patterns). Raw counts were transformed into log‐CPM (counts per million) values using the limma‐voom function to account for library size differences. The final differential expression analysis was performed using the lmFit function of the limma package to fit a linear model to the expression data for each gene. To identify differentially expressed genes (DEGs) relevant for our study, we included all pairwise and one‐vs‐rest contrasts of morphs (aquamarine vs. other groups, brown vs. other groups, green vs. other groups, red vs. other groups, aquamarine vs. brown, aquamarine vs. green, aquamarine vs. red, brown vs. green, brown vs. red, red vs. green). Moderated F‐statistics for testing whether all contrasts are zero, along with associated *p*‐values, were then computed for each gene, as implemented in the *eBayes* function, which stabilises variance estimates across genes and is particularly useful in small‐sample settings (Law et al. 2014). Resulting *p*‐values were adjusted for multiple testing using the Benjamini‐Hochberg false discovery rate (FDR) method (Benjamini and Hochberg 1995; Law et al. 2014). Genes were considered significantly differentially expressed (DEGs) if they had an adjusted *p*‐value < 0.05 (Benjamini‐Hochberg correction for multiple testing) and |log_2_ fold change| > 1. All identified DEGs between all comparisons performed are in Data S4.

To identify genes and DEGs linked to colour variation, we used an a priori list of colour‐related genes compiled by Monteiro et al. (2024). This list includes genes implicated in coloration, chromatophore development, and colour patterning across zebrafish, mice, humans and amphibians. The list includes 1020 genes categorised into five groups: carotenoid metabolism, guanine synthesis in iridophores, melanin synthesis, pteridine synthesis, and pigmentation variation (full list in Data S5).

Given that our chemical analyses of the skin revealed higher concentrations of polar carotenoids in the Red morph (see 3. Results), we screened all DEGs in the cytochrome P450 family as potential carotenoid ketolases. We then added the longest protein sequences of each of these *O. vicentei* candidates together with representative avian sequences of the *cyp2j19* gene to the alignment of Twomey, Johnson, et al. (2020); Twomey, Kain, et al. (2020) using maft (v7.31; Katoh and Standley 2013) with a BLOSUM62 substitution model and the “‐add, ‐keeplength, and ‐geneafpair (E‐INS‐i)” options. The resulting alignment of 1125 sites and 403 terminals was used for phylogenetic inference with iQTree v2 (Minh et al. 2020) after selecting the best‐fit nuclear amino acid model under the Bayesian information criterion (BIC). Node support was evaluated with 1000 ultrafast bootstrap pseudo‐replicates with nearest neighbour interchange.

The transcript sequences of *O. vicentei* with phylogenetic and expression profile similarity to existing ketolases were assessed for potential ketolase activity using a molecular docking experiment in the Swissmodel server (Bienert et al. 2017). Human cytochrome P450 3A4 (PDB ID 5VCC, 56% sequence identity) was selected as the template sequence (Uwangue et al. 2025). Other human cytochrome P450 3A4 with ligands crystallised structures were also used to improve the model. The homology model of *O. vicentei* ketolase together with the heme group of the human cytochrome P450 3A4 was used as the target and the ligand structure of β‐carotene was retrieved from PubChem and prepared using the Open Babel toolkit (O'Boyle et al. 2011). The ligand was energy‐minimised and converted to the MOL2 format prior to submission. To improve docking accuracy, the attracting cavities scoring function was used (Bugnon et al. 2024; Röhrig et al. 2023). This feature guided the docking algorithm toward the known binding pocket of the ketolase by biasing the search toward favourable regions based on cavity detection. The dimensions of the box were 20 × 20 × 20 Å.

### 2.10 Gene Co‐Expression Networks Analysis

Weighted Gene Co‐expression Network Analysis (WGCNA) was performed using the R package WGCNA v1.73 (Langfelder and Horvath 2008) to identify co‐expressed gene modules and key regulatory hub genes. Hub genes were identified as those with the highest intramodular connectivity within each module using the *chooseTopHubInEachModule* function. We used the TMM‐log transformed and low‐expression filtered gene counts from the limma analysis to construct a signed co‐expression network for each tissue separately. Pearson's correlation coefficients were used to estimate co‐expression strength, and a soft‐thresholding power transformation emphasised strong correlations (|*r*| > 0.8, *p* < 0.05). The soft‐thresholding power was selected using the *pickSoftThreshold* function in WGCNA by identifying the lowest power at which the scale‐free topology fit index (signed *R* ^2^) exceeded 0.80, indicating an approximate scale‐free network structure and was set at 7 for skin and 5 for liver. The data were then converted into an adjacency matrix, and a dissimilarity measure was used to assess gene connectivity clustering genes hierarchically into modules of similar expression profiles. Expression eigenvalues per module were correlated with four morph variables (aquamarine, brown, green, red), each coded as a binary (presence/absence) variable.

### 2.11 GO Enrichment Analysis

Functional enrichment analysis of DEGs and WGCNA modules was performed using the Over‐Representation Analysis (geneORA) function in the genekitr V1.2.9 R package (Liu and Li 2023) to identify overrepresented Gene Ontology (GO) terms including Biological Processes (BP), Molecular Functions (MF), and Cellular Components (CC). The GO reference sets were retrieved using the geneset::getGO function for human annotations. Fold enrichment was used to prioritise the degree of overrepresentation relative to the background gene set (i.e., all genes expressed in the tissue). For the DEGs, the main expression clusters were identified by hierarchical clustering (Ward's method) on the TMM‐log transformed and low‐expression filtered gene counts for skin and liver tissue separately. The number of clusters was determined by visual inspection of the dendrogram. For interpreting the functionality of WGCNA modules, a separate ORA was conducted for each relevant module.

### 2.12 Cross‐Species Comparison of Colour Related Genes in Dendrobatidae

To put our findings into perspective, we estimated the extent of overlap between our list of DEGs in *O. vicentei* and those from published transcriptomic studies involving divergent colour morphs or distinctly coloured skin patches in six other species of Dendrobatidae (Stuckert et al. 2019; Rodríguez et al. 2020; Twomey, Johnson, et al. 2020; Twomey, Kain, et al. 2020; Stuckert et al. 2023; Rubio et al. 2024). We annotated the resulting list of genes with the pigmentation roles from our list of colour‐related genes. To identify the level of conservatism in the gene expression profiles across species, we also quantified the overall percent of shared DEGs (those detected in two or more species across the different studies) and the number of shared DEGs for each combinatorial level of species (2–7). This approach should rank the list of DEGs in levels of conservatism across species. In these calculations, we also quantified the number of genes previously identified as colour related.

## 3 Results

### 3.1 Colorimetric, Histological and Carotenoid Content Variables

**FIGURE 1:**
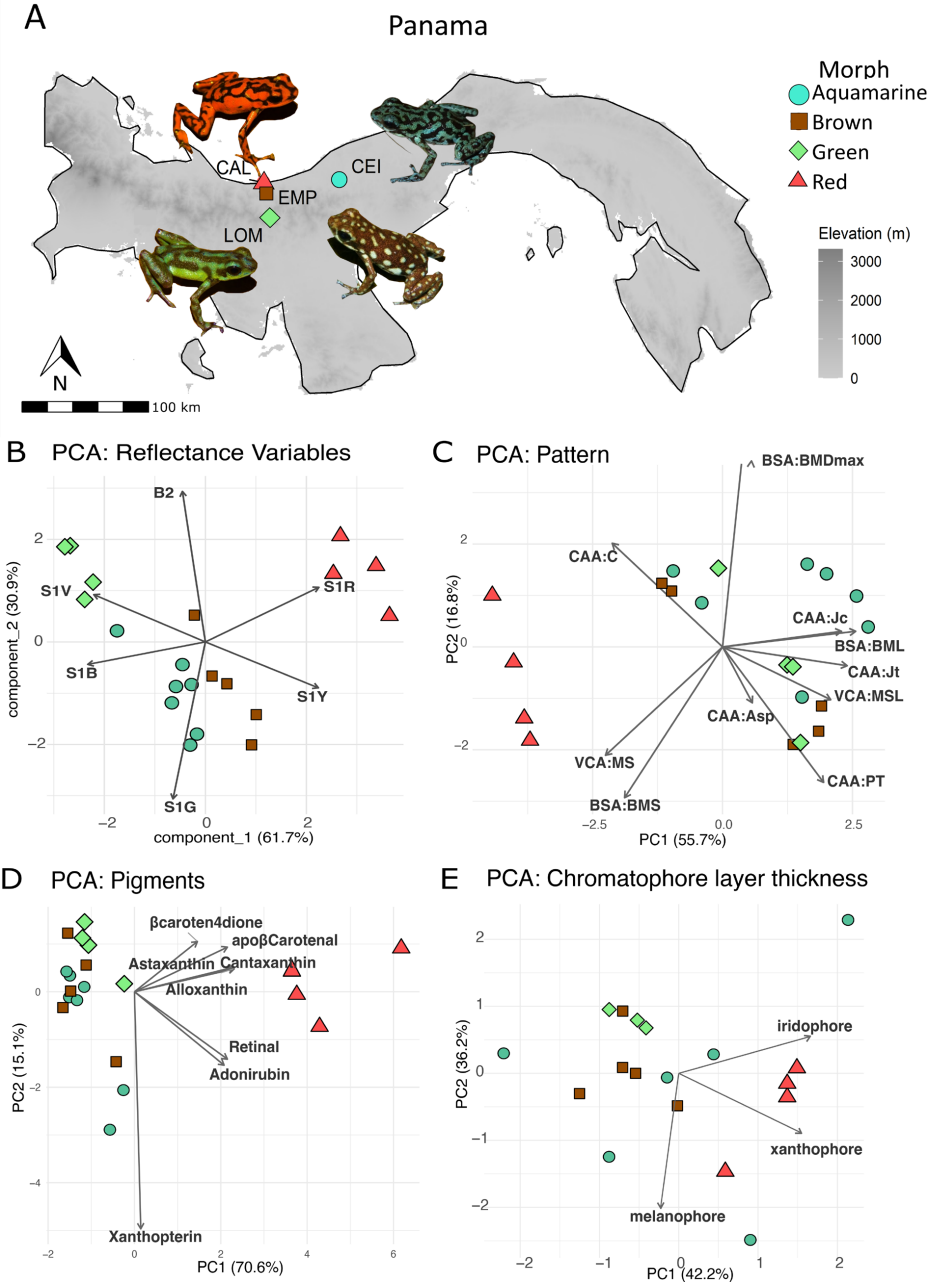
Sampling localities and phenotypic description of the four colour morphs of *O. vicentei* studied here. (A) Topographic map showing the sampling localities and a photograph of a representative frog for each locality: Calovébora, Veraguas Province (red morph, CAL); La Ceiba, Colón (aquamarine morph, CEI); Loma Grande, Veraguas Province (green morph, LOM); Empalizada, Veraguas Province (brown morph, EMP). (B–D) Principal component analyses (PCA) of colour and pattern traits across *Oophaga vicentei* morphs. (B) Spectral variables estimated by reflectance curve summary. (C) Pattern variables. (D) Skin pigment concentrations. (E) Chromatophore layer thickness. Morphs are shown by colour and shape; arrows indicate loadings of individual variables contributing to PCA axes; their direction and length represent variable correlation and contribution.

We identified an *O. vicentei* colour morph on each of the studied localities: Aquamarine (CEI), Brown (EMP), Green (LOM), and Red (CAL; Figure 1A). These colour morphs differed in spectral reflectance, with the red and green morphs being the most discriminable, located in opposing positions along the PC1 axis (most heavily loaded by S1R, S1Y, S1V, and S1B; Figure 1B; PCA loadings per variable on SM6). The aquamarine and brown morphs occupied an intermediate position between green and red and were less divergent from each other in the ordination space. Inspection of the reflectance curves showed that although the red morph showed a pronounced peak in the red wavelength region of the spectrum, the green morph displayed two peaks (in the violet and the green wavelength regions), resulting in similar mean spectral brightness values in these two morphs (Figure A1).

In terms of their colour patterns, Aquamarine frogs had greenish‐blue dorsa with black vermiculations; Brown frogs had dark brown dorsa with creamy‐yellow spots; Green frogs showed green dorsa with dark bands and Red frogs displayed red dorsa with dark bands. The individual scores on the PCs derived from the 10 colour pattern variables showed a clear distinction of the Red morph from the other three morphs along the first PC (most heavily loaded by CAA.Jc, CAA.Jt, and BSA.BML) but an overlap between the Aquamarine, Brown, and Green morphs, which could not be distinguished in ordination space (Figure 1C).

Red frogs showed the highest concentrations of keto‐carotenoids and apocarotenoids like cantaxanthins and apo‐β‐carotenal. Xanthophylls were also more abundant in red frogs compared to the other three morphs. Xanthopterin, a pteridine, was present across all morphs, with the highest mean concentration in aquamarine individuals. Total polar carotenoids concentration was higher for red individuals compared to other morphs. PCA analysis revealed that the first two components explained 70% of total variation with the higher loadings on PC1 and PC2 from xanthopterin, ketocarotenoids (Adonirubin, Astaxanthin, Alloxanthin, and Cantaxanthin) and apo‐β‐carotenal. The red frogs were the more differentiated, driven by xanthopterin and astaxanthin (Figure 1E, Figure A2, and Table 2).

**TABLE 2:** Summary of pigment compounds identified in *Oophaga vicentei* morphs (aquamarine, brown, green, red) and mean estimation of concentration per morph. Columns include; Compound: Abbreviated chemical name of the pigment; A: Aquamarine; B: Brown; Class: Chemical classification in accordance to the results and discussion text; Conc.: Concentration (ng/g), mean ± standard deviation of pigment concentration in each colour morph; G: Green; Mol. F.: Molecular formula, chemical formula of the pigment; R: Red.

Compound	Class	Mol.F.	A conc. (ng/g)	B conc. (ng/g)	G conc. (ng/g)	R conc. (ng/g)
Xanthopterin	Pteridine	C_6_H_5_N_5_O_2_	238,678.09 (±288797.31)	147,038.04 (±117504.85)	50,567.08 (±15598.71)	109,051.15 (±55167.8)
Adonirubin	Ketocarotenoid	C_40_H_52_O_3_	1042.95 (±1167.93)	5583.66 (±4700.37)	727.7 (±740.89)	7742.1 (±6025.05)
Astaxanthin	Ketocarotenoid	C_40_H_52_O_4_	0 (±0)	0 (0)	0 (0)	88.33 (±74.58)
Canthaxanthin	Ketocarotenoid	C_40_H_52_O_2_	81.83 (±216.5)	0 (0)	0 (0)	926.56 (±559.85)
Retinal	Retinoid	C_20_H_28_O	8.22 (±5.71)	6.07 (±4.22)	7.62 (±4.56)	42.41 (±20.31)
apoBCarotenal	Apocarotenoid	C_22_H_30_O	0 (±0)	1.7 (±2.52)	5.66 (±3.64)	117.13 (±54.35)
BCarotene3ol	Xanthophyll	C_40_H_54_O	0 (±0)	0 (0)	0.3 (±0.39)	79.18 (±39)
CanXanthophyllB		C_40_H_52_O_2_	0 (±0)	0 (0)	0 (0)	0.72 (±1.43)

**FIGURE 2:**
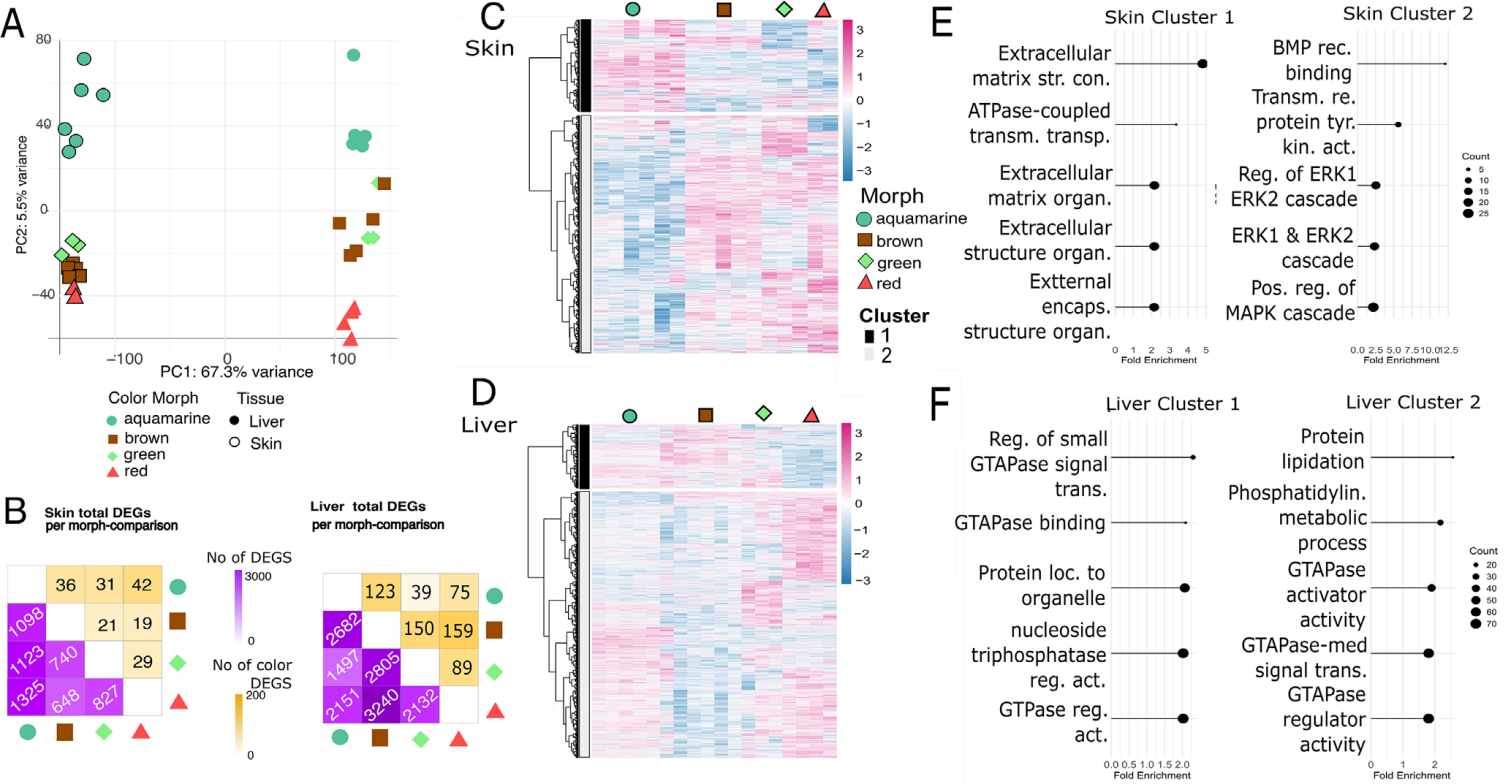
Differential Gene expression in Skin and Liver tissue across four colour morphs of *O. vicentei* . (A) PCA plot of gene expression variance across all samples, grouped by tissue type (skin and liver samples) and colour morph. Skin samples are highlighted with outlined symbols, liver samples are highlighted with non‐outlined symbols. Shape and colours indicate morph. (B) Matrix diagram showing the number of differentially expressed genes (DEGs) in skin (left) and liver (right) between each morph pairwise comparison. Bellow the diagonal the number of total DEGs are shown, above the diagonal the subset of DEGs that have been previously characterised as colour related are shown. (C) Heatmap of DEG expression patterns in skin samples. Rows: Genes (row‐scaled z‐scores); Columns: Samples grouped by morph. Heatmap shows morph‐specific expression profiles (blue = low, pink = high). Hierarchical clustering (Ward's D2) grouped genes into expression clusters, which reflect morph‐specific transcriptional profiles. Coloured bars above the heatmap indicate colour morph; coloured bars on the left indicate gene clusters. (Cluster 1: Genes highly expressed in aquamarine morph; Cluster 2: Genes highly expressed in red frogs). (D) Heatmap of DEG expression patterns in liver samples (as in panel C; Cluster 1: Genes highly expressed in aquamarine frogs; Cluster 2: Genes highly expressed in red frogs). (E) Functional enrichment of skin DEG clusters. Top Gene Ontology (GO) terms of BP shown by fold enrichment. (F) Functional enrichment of liver DEG clusters (as in panel D).

Bright field microscopy showed no significant differences in melanophore (*p* = 0.165) and iridophore (*p* = 0.242) thickness among morphs, but xanthophore thickness was significantly greater in red frogs (*p* = 0.023), confirmed by post hoc Tukey's test (*p* < 0.05 vs. Green and Brown; Table 3). Multivariate ordination of these three variables showed a clear distinction of the Red morph (some overlap with extreme aquamarine individuals) from the other three morphs, which overlapped in ordination space (Figures 1D and 2).

**TABLE 3:** Summary of statistical test results for chromatophore layer thickness across different colour morphs, including xanthophore, melanophore, and iridophore layers. Statistical results for one‐way ANOVA test for group mean differences among groups and Tukey's post hoc tests for pairwise comparisons when ANOVA assumptions were met are shown. For each variable, the table presents the mean thickness (μm), standard deviation (SD), the coefficient of variation (CV), the statistical test for mean differences between morphs. The F‐statistics (F) and associated degrees of freedom (df) for each ANOVA are reported in the table. Bolded *p*‐values indicate statistically significant differences (*p* < 0.05).

Variable	Morph	Mean (μm) ± SD, CV	Test	F (Df1, Df2)	*p*
Xanthophore thickness	Aquamarine	29.4 ± 5.75, 19.6%	Tukey (aq.–brown)		0.90
			Tukey (aq.–green)		0.94
			Tukey (aq.–red)		0.06
	Brown	27.2 ± 3.35, 12.3%	Tukey (brown–green)		0.99
			Tukey (brown–red)		**0.027**
	Green	27.3 ± 0.94, 3.4%	Tukey (green–red)		**0.043**
	Red	38.1 ± 4.47, 11.7%	ANOVA	4.209 (3, 16)	**0.023**
Melanophore thickness	Aquamarine	97.3 ± 37.80, 38.8%	ANOVA	1.93 (3, 16)	0.165
	Brown	81.0 ± 10.80, 13.3%			
	Green	57.8 ± 6.65, 11.5%			
	Red	80.0 ± 24.20, 30.3%			
Iridophore thickness	Aquamarine	26.2 ± 10.80, 41.3%	ANOVA	1.542 (3, 16)	0.242
	Brown	20.0 ± 1.51, 7.6%			
	Green	18.5 ± 2.90, 15.6%			
	Red	26.1 ± 4.89, 18.7%			

### 3.2 Transcriptome Assembly

A total of 44 transcriptome assemblies were generated across four localities. BUSCO assessment revealed a high level of completeness for each individual assembler transcriptome. The final consensus transcriptome resulting from the Evidential Gene pipeline contained 883,936 transcripts (average length 1100 bp) and had 95.78% of the 3354 Benchmarking Universal Single‐Copy Orthologs (BUSCOs) identified as complete and was superior to the input assemblies, ensuring its suitability as a reference for downstream analysis (see Figure A3 and Table A2).

### 3.3 Differential Gene Expression Analysis

A total of 38,863 genes (285,382 transcripts) were annotated in our de novo transcriptome assembly. PCA showed tissue type as the main source of transcriptomic variation, with skin and liver samples clearly separated along PC1 (68% variance). Within tissues, colour morphs varied along PC2 (5%), with the aquamarine morph diverging most strongly, particularly from red frogs (Figure 2A). After expression filtering, we retained 14,829 genes from skin and 13,560 from liver for DGE analyses.

We identified 1838 DEGs in skin and 5085 in liver across at least one pairwise comparison among the four morphs (Figure 2B). Most DEGs were between aquamarine and red frogs in both tissues. In skin, Cluster 1 genes (Figure 2C, dark grey annotation) were overexpressed in aquamarine frogs. Top enriched terms for cluster 1 include BP related to extracellular‐matrix (ECM) and transmembrane transportation. Genes of cluster 2 in skin are associated with BMP binding, ERK1/ERK2 and MAPK cascade regulation (Figure 2E). In liver, cluster 1 genes were lowly expressed in red frogs (Figure 2D, dark grey annotation) and are associated with GTPase binding and regulation. Genes of cluster 2 are associated with protein lipidation, GTPase activity, and small GTPase transport (Figure 2F; Full enrichment list on SM7).

**FIGURE 3:**
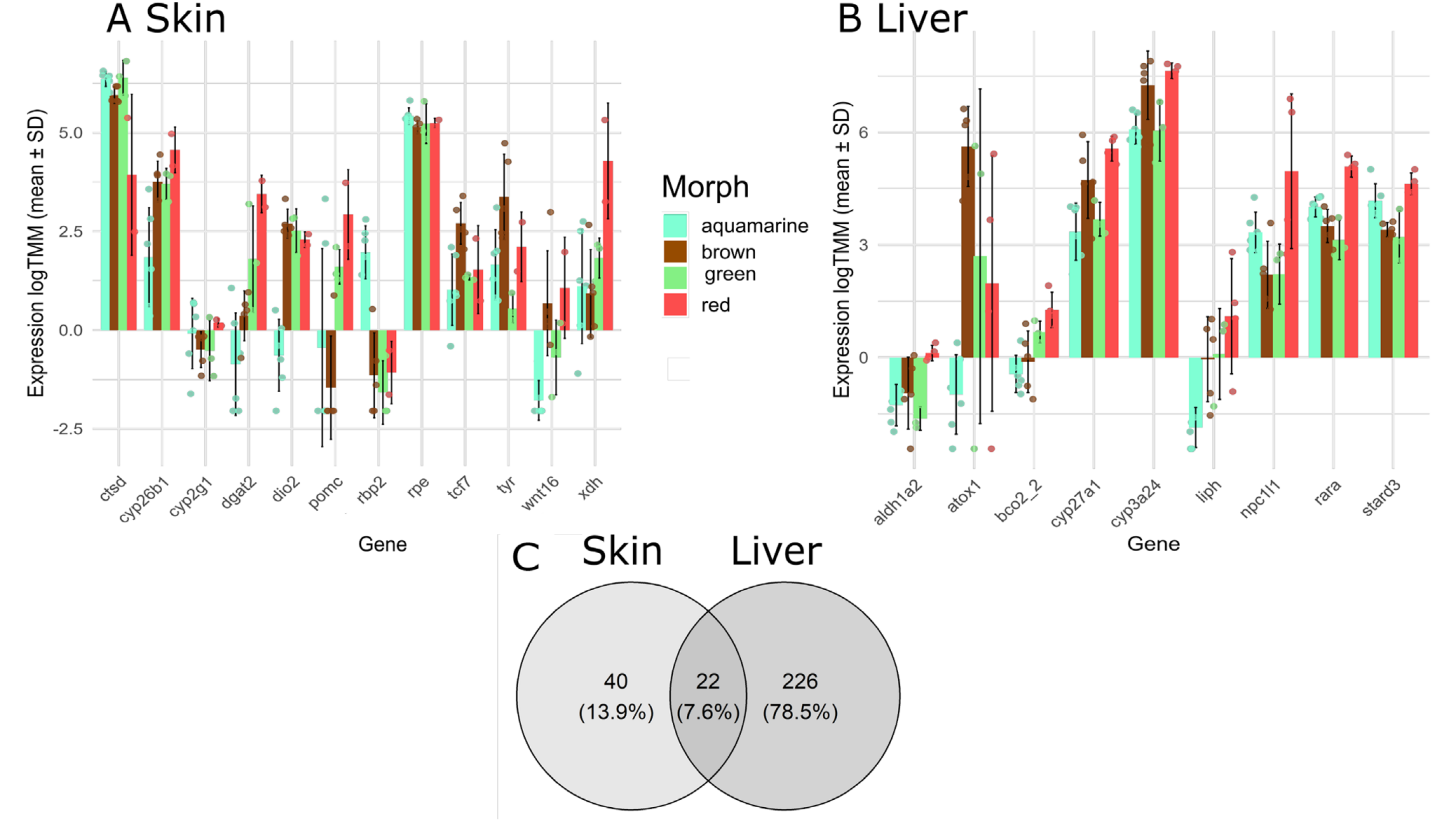
Bar plots showing expression levels of selected pigmentation‐related DEGs in skin (A) and liver (B) across four colour morphs: Aquamarine, brown, green and red. DEGs were selected based on significant differential expression (|logFC| > 1.0, *p* < 0.05). Genes of known pigmentation function are selected and shown here. Bars represent normalised expression (e.g., log‐transformed TMM counts). (C) Venn diagram summarising colour‐related DEGs exclusive to skin, exclusive to liver, or shared between both tissues. See Figures A3 and A4 and Table S2 for full DEG lists and comparisons.

We found 74 colour‐related DEGs in skin and 267 in liver. In skin, 11 colour‐related DEGs were involved in carotenoid metabolism, 13 to melanin synthesis, 2 to guanine synthesis in iridophores, and 1 to pteridine synthesis. An additional 47 DEGs were linked to pigmentation variation (Figure 3A). In liver, we found 30 melanin‐related DEGs, 14 carotenoid‐related, 8 guanine‐related, 7 pteridine‐related, and 207 linked to pigmentation variation (Figure 3B and Table 4). In total, 22 colour‐related genes were shared between tissues (14.19% of all colour DEGs), 40 unique to skin, and 226 to liver (Figure 3C).

**TABLE 4:** Differentially expressed genes (DEGs) related to pigmentation in the skin and liver tissues of *Oophaga vicentei* colour morphs. Listed genes were identified as DEGs with known roles in pigmentation and are presented alongside the tissue and morph(s) in which differential expression was detected. Arrows indicate the direction of regulation (↑ upregulated, ↓ downregulated) relative to other morphs. Gene functions are based on previously published studies of pigmentation in polymorphic vertebrate species.

Gene	Tissue	Morph	Function	Source
*tyr*	Skin	Aquamarine↓, Brown↑	Melanin synthesis	McLean et al. (2017)
*pomc*	Skin	Green↑	Melanogenesis (α‐MSH production)	McLean et al. (2017)
*tcf7*	Skin	Brown↑	Promotes melanophore development	McLean et al. (2017)
***wnt16***	Skin	Brown↑, Red↑	WNT signalling, pigment cell regulation	McLean et al. (2017)
***dgat2***	Skin	Red↑	Carotenoid storage	McLean et al. (2017)
***cyp26b1***	Skin	Aquamarine↓, Red↑	Retinoid degradation and signalling	McLean et al. (2017)
*rbp2*	Skin	Aquamarine↑	Retinoid transport	McLean et al. (2017)
***rpia***	Skin	Brown↑	Guanine synthesis (iridophores)	Higdon et al. (2013)
*dio2*	Skin	Aquamarine↓	Melanophore regulation	Baxter et al. (2019)
**ctsd**	Skin	Red↓	Melanin degradation	Baxter et al. (2019)
*npc1l1*	Liver	Red↑	Carotenoid absorption	McLean et al. (2017)
*aldh1a2*	Liver	Red↑	Retinoic acid production	McLean et al. (2017)
*bco2*	Liver	Red↑	Carotenoid degradation	McLean et al. (2017)
*rara*	Liver	Red↑	Pigment metabolism regulation	McLean et al. (2017)
*stard3*	Liver	Red↑	Carotenoid transport/storage	Bandara and von Lintig (2022)
*liph*	Liver	Aquamarine↓	Lipid metabolism, carotenoid transport	Baxter et al. (2019)
*xdh*	Skin, Liver	Red↑, Green↑	Pteridine synthesis	McLean et al. (2017)

**FIGURE 4:**
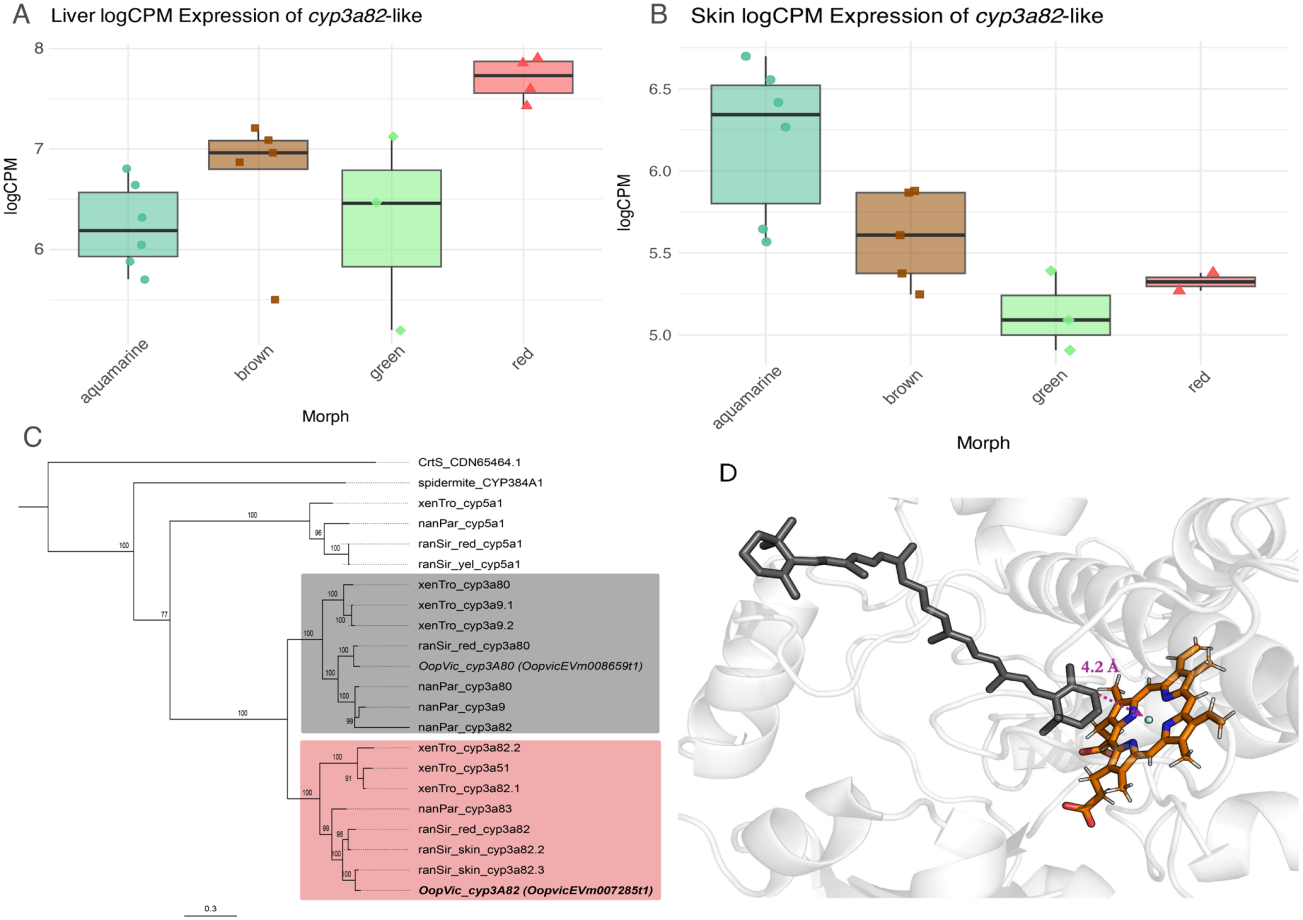
Expression profile, phylogenetic position and predicted β‐carotene C4 ketolation activity of the *Oophaga vicentei* ketolase candidate *cyp3a82*. Expression profile in liver (A) and skin (B); ***C*** *Maximum* likelihood phylogenetic tree of anuran cytochrome p450 sequences in the 3A family and closely related proteins (from Twomey, Johnson, et al. (2020); Twomey, Kain, et al. (2020)) together with *O. vicentei* sequences generated in this study (italics). The alignment includes yeast astaxanthin synthase (*CrtS*), spidermite (*cyp384A1*), *Xenopus tropicalis* (xenTro), *Nanorana parkeri* (nanPar), and *Ranitomeya sirensis* (ranSir) sequences. The maximum likelihood inference tree was obtained using a JTT + F + R8 protein substitution model (best fit according to its BIC) with node support evaluated with 1000 ultrafast bootstrap pseudo‐replicates. The clades containing known ketolase candidates of poison frogs are shaded: *R. sirensis* ketolase (*cyp3a80*, grey) and *O. vicentei* (*cyp3A82*, red). Note that an orthologous to the *R sirensis* ketolase exists in *O. vicentei* but shows no differential expression between colour phenotypes. (D) Structural homology model of the *cyp3a82*‐like enzyme showing the predicted pose of β‐carotene (black molecule) within the active site of the enzyme (light grey: Protein, orange: Heme prosthetic group). The β‐ionone ring's C4 atom is located approximately 4.2 Å (purple arrow) above the heme prosthetic group, a distance consistent with catalytic activity for C4 ketolation. This spatial arrangement and the favourable docking score (−8.25 kcal/mol) support the hypothesis that *cyp3a82* may function as a ketolase, although direct functional validation would substantially support the in silico results. The *β*‐ionone C4 atom in β‐carotene resides over the heme prosthetic group to ~4.2 Å (purple arrow) which positions it adequately for subsequent C4 ketolation.

A *cyp3*‐like gene in *O. vicentei* (named *cyp3a24* in Data S4) was upregulated in the liver and downregulated in the skin of red frogs (Figure 4A,B). Phylogenetic analysis placed the longest transcript sequence of this gene (OopvicEVm007285t1) within the well‐supported *cyp3A82* clade, closely related to the *cyp3A80* clade that includes the *R. sirensis* ketolase candidate (Figure 4C). Phylogenetic analysis placed it within the well‐supported *cyp3A82* clade, closely related to the *cyp3A80* clade that includes the *R. sirensis* ketolase candidate (Figure 4C and Figure A4 and Data S2). Docking with β‐carotene yielded a binding score of ~8 kcal/mol, with the C4–H bonds of the β‐ionone ring ~4.2 Å above the heme group, favouring C4‐ketolation (Figure 4D). The best homology model showed a QMEANDisCo global score of 0.80 ± 0.05, and the conserved AGYETTS motif along with substrate‐stabilising residues Ala310 and Thr314 further support its functional similarity to human CYP3A4 (Data S2 and Figures A5 and A6).

### 3.4 Gene Co‐Expression Networks Analysis

**FIGURE 5:**
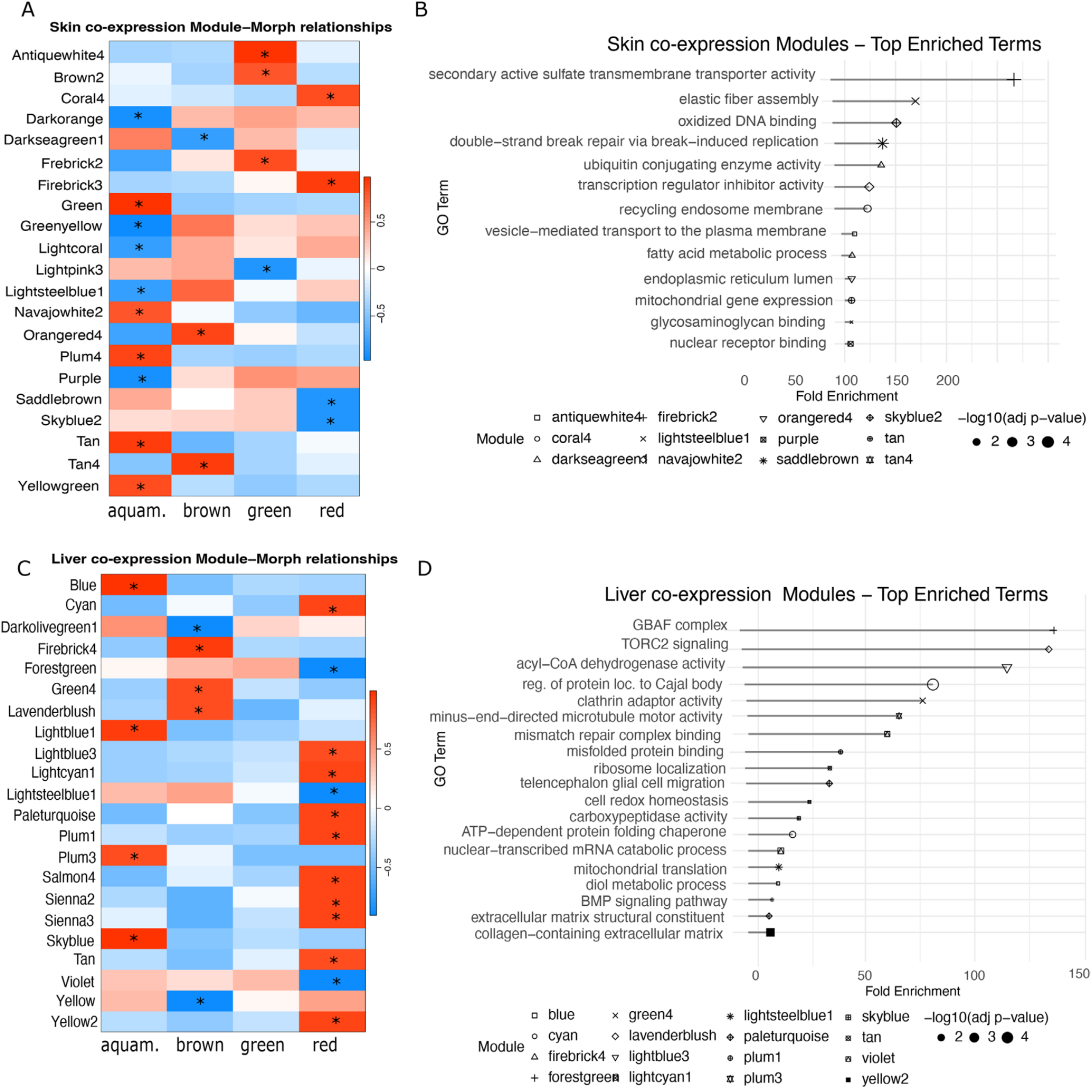
Gene co‐expression modules correlated with each of the four *O. vicentei* morphs studies here (aquamarine, brown, green, red) and their functional enrichment. (A) Module‐morph correlations for skin. Each row represents a gene co‐expression module (labelled by colour name), and columns represent morphs (Aquamarine, Brown, Green, Red). Colour intensity indicates the Pearson correlation coefficient (Cor), with red for positive and blue for negative correlations. Strong, statistically significant correlations (*r* ≥ |0.8|, *p* < 0.05) are annotated with asterisk (*). (B) Enriched Gene Ontology (GO) biological process (BP) terms for skin‐associated modules. Terms are listed with their respective modules (symbol) and adjusted *p*‐values (dot size). (C) Module‐trait correlations for liver, formatted identically to A. (D) Enriched GO terms for liver‐associated modules, formatted as in B.

In skin, we identified 177 gene co‐expression modules, 21 of which correlated significantly with at least one morph (Figure 5A). Ten modules were associated with the aquamarine morph and 3–4 to each of the other three morphs. In liver, 322 modules were detected, with 22 showing significant eigengene‐morph correlations (Figure 5C): 13 linked to the red morph, 4 to aquamarine and 5 to brown; none to green. Morph‐related modules of both tissues included many DEGs (Table 5), but few coloration‐related genes. Functional enrichment revealed 12 skin morph‐related modules enriched for GO terms such as collagen containing extracellular matrix, BMP signalling pathway, mitochondrial translation and TORC2 signalling (Figure 5B). Sixteen liver morph‐related modules were enriched for processes including vesicle mediated transport, metabolic process, double‐strand break repair and transmembrane transporter activity (Figure 5D). Full lists of enriched terms for all modules are provided in SM8. The ketolase candidate gene *cyp3a82*‐like clustered in the skin module plum4 (positively correlated with the aquamarine morph) and liver module mistyrose4 (not correlated with any colour morph) (Table 5; gene‐membership per module on Data S9).

**TABLE 5:** Gene co‐expression modules in skin and liver tissues of *O. vicentei* that show strong correlations with colour morph traits. Each module is named after a unique colour and is associated with a hub gene, a central regulatory gene within the module. Bolded hub genes indicate colour‐related DEGs. For each module, the table shows the total number of genes, number of DEGs, number of colour‐related genes, and number of colour‐related DEGs. Abbreviations: Hub G., hub gene; Total G., total number of genes; DEGs, number of differentially expressed genes; Colour G, number of colour‐related genes; Colour DEGs, number of colour‐related DEGs.

Tissue	Module	Total G.	DEGs	Colour G.	Colour DEGs
Skin	antiquewhite4	67	25	7	2
	brown2	55	7	4	0
	coral4	36	15	0	0
	darkorange	135	51	6	2
	darkseagreen1	25	5	1	0
	firebrick2	31	21	0	0
	firebrick3	42	26	2	1
	green	377	89	13	2
	greenyellow	281	128	14	5
	lightcoral	56	29	1	1
	lightpink3	50	6	2	0
	lightsteelblue1	95	54	2	2
	navajowhite2	77	25	0	0
	orangered4	96	39	2	1
	plum4	39	12	2	0
	purple	295	121	8	2
	saddlebrown	123	36	9	4
	skyblue2	64	9	6	0
	tan	253	39	5	1
	tan4	39	15	0	0
	yellowgreen	99	12	1	0
Liver	blue	378	230	12	7
	cyan	126	83	7	4
	darkolivegreen1	33	15	2	1
	firebrick4	47	17	3	2
	forestgreen	14	11	0	0
	green4	35	12	1	0
	lavenderblush	31	17	1	0
	lightblue1	26	13	2	1
	lightblue3	33	19	2	1
	lightcyan1	63	40	5	5
	lightsteelblue1	64	42	5	3
	paleturquoise	71	44	0	0
	plum1	65	41	5	4
	plum3	46	33	2	2
	salmon4	55	36	6	5
	sienna2	33	24	0	0
	violet	69	40	3	2
	yellow	296	112	11	4
	yellow2	33	17	1	1

### 3.5 Cross‐Species Comparison

**FIGURE 6:**
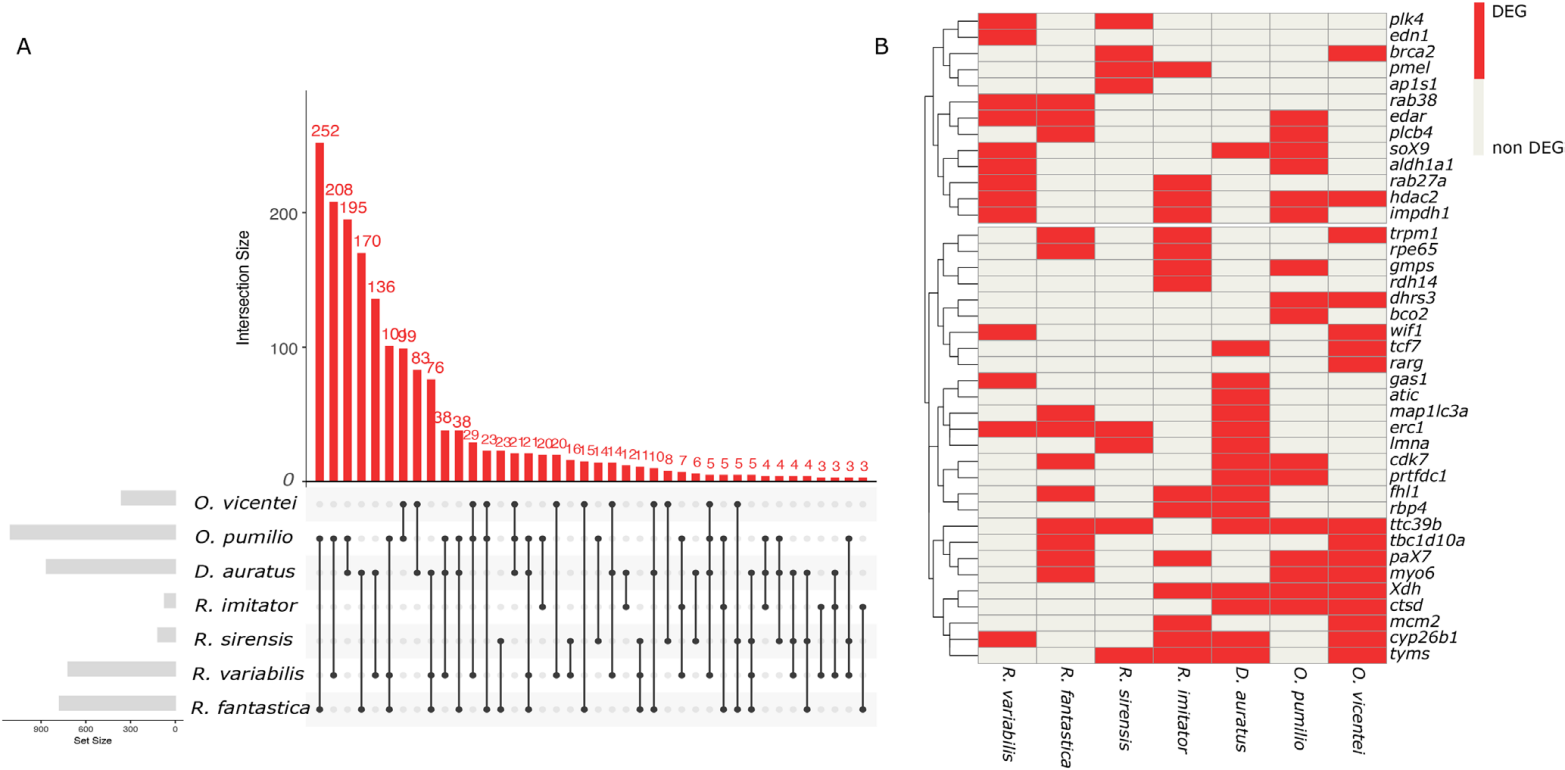
Comparative analysis of DEGs between colour morphs of *O. vicentei* and similar studies in other six dendrobatid species. (A) The UpSet plot visualises total shared DEGs between colour morphs across the seven species with the top bar plot representing the number of shared genes in each comparison. (B) A dendrogram of a subset of colour related DEGs identified in two or more species. See methods for the references of each species. Each row represents a gene; each column corresponds to a species. To enhance visual clarity, a representative subset of the full set of colour‐related DEGs is shown. Colour key: Red = DEG present, light grey = DEG absent.

Our compilation includes 3993 differentially expressed genes (DEGs) from skin tissue across colour morphs or skin colour patches of seven dendrobatid species, including this study (Figure 6). Inspection of this compilation highlights 360 of the DEGs in *O. vicentei* (25 of them colour‐related) that have been previously found in comparable studies in one to four other species of Dendrobatidae. Family wide, 43% of the DEGs were shared between two or more species but no DEGs were common to all seven species. Five DEGs were shared among five species, but only one of these genes (*ttc39b*) is known to be colour related. Another 44 DEGs were shared among four species; seven of these have been previously suggested as colour‐related (*cyp26b1*, *tyms*, *hdac2*, *pax7*, *erc1*, *ric8b*, *xdh*). Many more DEGs were shared in combinations including three (412 DEGs, 40 colour‐related) or two (1286 DEGs, 102 colour‐related) species (Figure 6; full list of common DEGs on Data S10). Our results also identify 1597 DEGs with no known role in vertebrate coloration but significantly associated with colour differences in two to five species of Dendrobatidae.

## 4 Discussion

Our analysis reveals a clear differentiation of the red *O. vicentei* morph from the aquamarine, brown, and green morphs in spectral reflectance, colour pattern, chromatophore thickness, and pigment composition. Red frogs accumulate more keto‐carotenoids and have a thicker xanthophore layer, while the other three morphs overlap in phenotypic traits, suggesting structural coloration or undetected pigments might drive colour variation. Red frogs upregulate genes for carotenoid metabolism, i.e., there are active pathways for red pigmentation in both skin and liver. Aquamarine frogs showed the most divergent transcriptional profiles despite their overall phenotypic overlap with brown and green frogs. This pattern could result from the genetic divergence of this morph from the other three given its more isolated geographic location. Overall, red coloration in this species seems to derive from ketocarotenoid bioaccumulation in the skin, while the colouration differences among the other three morphs likely result from structural coloration or unknown pigments.

### 4.1 Chromatophore Organisation and Carotenoid Accumulation Underlying Red Coloration

In *O. vicentei* , red frogs show less variation between their dorsal background colour and the dark vermiculations, but higher contrast, which may enhance their detectability and aposematic signalling against diverse backgrounds (Troscianko et al. 2017). Red frogs also have a thicker xanthophore layer, which explains the higher concentration of carotenoids.

In contrast, aquamarine, brown, and green frogs show no significant differences in skin chromatophore layers, and pigment profiles are roughly similar across these three morphs. The relatively small sample sizes of our study may limit statistical power to detect subtle phenotypic or molecular differences. The lack of differences in the concentrations of the pigment classes suggests that coloration differences in these morphs may either derive from pigments not detected by our assays or most likely by structural coloration differences resulting from the scattering of light on melanin, xanthopterin, iridophore guanine platelets, and collagen fibres, and the interaction of those components (Bagnara et al. 2007; Twomey, Johnson, et al. 2020; Twomey, Kain, et al. 2020; Rojas et al. 2023).

### 4.2 Transcriptomic Basis of Colour Divergence

Red coloration in vertebrates is typically carotenoid‐based, involving carotenoid absorption from dietary sources, transport, and cleavage by *bco1*/*bco2* enzymes (Lobo et al. 2012; Twomey, Johnson, et al. 2020; Twomey, Kain, et al. 2020). In *O. vicentei* , red frogs upregulated *dgat2* in the skin, which promotes carotenoid accumulation (Ahi et al. 2020), and *cyp26b1*, an enzyme involved in retinoid metabolism (Ocaya et al. 2010). A candidate ketolase, *cyp2g1*, was also upregulated in red skin, paralleling patterns in *Ranitomeya sirensis* (Twomey, Johnson, et al. 2020; Twomey, Kain, et al. 2020). Additionally, our results suggest that in the liver of the red frogs a *cyp3a82*‐like enzyme may catalyse the C4‐ketolations leading to elevated levels of ketocarotenoids in these frogs. Our findings also suggest that the ketolase in *O. vicentei* is distinct from the one in *R. sirensis* and point to the independent evolution of this key aposematic trait in different lineages of Dendrobatidae.

The *cyp3a82*‐like *gene* was DE in skin but downregulated in red frogs, suggesting functional substitution by other CYP enzymes such as *cyp2g1* (Twomey, Johnson, et al. 2020; Twomey, Kain, et al. 2020). Alternatively, it may be acting primarily in the liver, where carotenoid metabolism occurs, or antagonise carotenoid accumulation in skin. Such patterns support the hypothesis that redundant, independently co‐opted enzymatic pathways contribute to red phenotypes (Twomey, Johnson, et al. 2020; Twomey, Kain, et al. 2020).

Along with the *cyp3a82* ketolase, the *npc1l1, aldh1a2, rara* and *stard3* genes involved in enhanced carotenoid processing (During et al. 2005; Johnson and Hill 2013; Sluchanko et al. 2022) were also upregulated in the liver of red frogs. Additionally, *xdh*, a key enzyme in pteridine synthesis, is highly expressed in both skin and liver of red frogs. This gene has been widely reported as differentially expressed and associated with pteridine‐based coloration across colour morphs of dendrobatids and other taxa (Rodríguez et al. 2020; Aguilar‐Gómez et al. 2024; Rubio et al. 2024; Stuckert et al. 2019; Braasch et al. 2007; McLean et al. 2017), suggesting its conserved role in colour variation. Overall, we detected more DEGs in the liver than in the skin, highlighting the diversity of the liver's functions and its likely role in metabolising pigments before transportation to the skin, as observed in passerine birds (del Val et al. 2009; Andrade et al. 2019).

Comparatively, aquamarine frogs showed the most divergent transcriptional profiles, possibly reflecting distinct regulatory mechanisms or population‐specific divergence and evolutionary distance as this locality is geographically more isolated from the rest. Aquamarine‐upregulated genes in skin were enriched for ECM organisation and ATPase‐coupled transport, processes that might influence chromatophore positioning and intracellular trafficking (Erickson 1993; Thibaudeau and Altig 2012; Tucker and Erickson 1986; Sköld et al. 2012). Conversely, BMP and MAPK pathway genes (and particularly the *tyr* gene, essential for melanin production) were downregulated in the aquamarine morph, reflecting reduced melanogenesis (Bertrand et al. 2009). Together, these patterns support the role of structural coloration mechanisms in amphibian skin colour variation.

### 4.3 Gene Co‐Expression Modules

Co‐expression analysis indicates that colour variation in *O. vicentei* is shaped by tissue‐specific gene networks rather than single genes. In skin, most morph‐related modules are associated with the aquamarine morph, whereas in liver, most morph‐related modules are associated with the red morph. These findings suggest that aquamarine coloration is primarily driven by pathways active in the skin, whereas red coloration depends more on liver‐associated processes.

Only two aquamarine‐related modules in skin are enriched. These two modules (Lightsteelblue1, Navajowhite2) are enriched for Ubiquitin activity and DNA repair, and Elastic fibre assembly and oxidised DNA binding, respectively. Elastic fibre assembly contributes to skin structure and elasticity and may influence the final coloration through structural coloration (Bagnara et al. 2007; Sköld et al. 2012). Lightsteelblue1, while less directly linked to coloration, could have an indirect effect through cellular integrity maintenance.

Red coloration is associated with carotenoid pigments and the red‐related modules in liver are enriched for metabolic and trafficking processes of these pigments. Module cyan is enriched for mitochondrial translation which influences cellular metabolism and energy balance, suggesting higher metabolic needs for red coloration. This is observed in other vertebrates as well (del Val et al. 2009). Red‐related modules are associated with acyl‐CoA dehydrogenase activity, a key enzyme in fatty acid β‐oxidation and may indirectly influence carotenoid‐based pigmentation by supporting lipid transport and energy metabolism required for pigment deposition. Module plum1 is enriched for BMP signalling, a function directly involved in pigment cell differentiation and tissue patterning, influencing both pigment synthesis and structural coloration.

Many morph‐related modules in both skin and liver lacked functional enrichment yet contained DEGs and some colour‐related genes, a pattern consistent with findings in other species where coloration genes often participate in broader regulatory networks (Twomey, Johnson, et al. 2020; Twomey, Kain, et al. 2020). These modules may harbour unexplored genes involved in pigment metabolism or structural coloration and thus represent promising targets for future functional exploration.

### 4.4 Cross‐Species Comparison

Our comparison of DEGs between colour morphs in seven dendrobatid species revealed a core group of DEGs that may play conserved roles across species in the family. In this group, the *ttc39b* gene is known to enhance ketocarotenoid production in birds (Toomey et al. 2022) and has recently been associated with yellow/red skin colour polymorphism in *O. pumilio* (Aguilar‐Gómez et al. 2024). Differences in phenotypes, sampling sizes, environment, or population structure complicate direct comparisons of regulatory mechanisms across species. These findings provide a baseline for prioritising DEGs in future studies in amphibians and offer a framework for comparative transcriptomics analysis of colour polymorphism or polytypism.

### 4.5 Conclusion

Colour polytypy in *O. vicentei* results from a complex interplay of chromatophore architecture, pigment composition, and gene expression. Red frogs have a thicker xanthophore layer and high concentrations of keto‐carotenoids. These red morph traits are associated with an upregulation of genes involved in carotenoid accumulation, retinoid metabolism, and a candidate ketolase distinct from those described in other dendrobatids, pointing to an alternative enzymatic pathway for C4‐ketolation. In contrast, aquamarine, brown, and green morphs share low carotenoid diversity, implying that structural coloration or alternative pigments contribute to these phenotypes. Aquamarine frogs show transcriptional signatures of reduced melanogenesis and enrichment for ECM organisation and transport processes, supporting the role of structural coloration in this morph. Co‐expression analyses revealed morph‐specific modules of genes with no previously known pigmentation roles, emphasising the evolutionary lability and polygenic nature of amphibian skin coloration. The minimal overlap between the set of morph‐associated genes in *O. vicentei* and those of comparable studies in other dendrobatids suggests that aposematic phenotypes evolve through multiple, lineage‐specific genetic routes, combining conserved biochemical processes with novel regulatory mechanisms.

## Author Contributions

V.M.‐O. and A.R. designed the study. V.M.‐O., R.I., and A.R. collected samples. V.M.‐O. and A.R. performed methodology development. V.M.‐O. performed RNA extractions. V.M.‐O. and A.R. conducted transcriptome assembly and bioinformatics analysis. V.M.‐O. wrote the original manuscript draft, with critical revisions contributed by A.R., G.T.‐C., R.I., W.J.Z.R., and H.P. A.R., G.T.‐C., and H.P. secured funding and supervised the project.

## Funding

Financial support for this project included DFG grants to A. Rodríguez (RO5508/2‐1) and Heike Pröhl (PR 626/10‐1), as well as funding from the Sistema Nacional de Investigación and the Panama Amphibian Rescue and Conservation project to R. Ibáñez. G. Tamayo‐Castillo’s participation was made possible by the CONARE‐DFG grant (No. FS‐DFG‐01‐2022), coordinated by El Consejo Nacional de Rectores (CONARE) and by the Vicerrectory for Research of the University of Costa Rica (809‐C2‐654).

## Disclosure

*Data accessibility statement*: The raw sequencing reads for each sample (accession numbers: SRR32735198—SRR32735179) are available in the NCBI Sequence Read Archive (SRA) under the Bioproject PRJNA1234489. This Transcriptome Shotgun Assembly project has been deposited at DDBJ/EMBL/GenBank under the accession GLHG01000000. All bioinformatic analysis scripts used in this study are publicly available on GitHub at github.com/BillieMantzOik/VICTRA. Asociated metadata can be found in github.com/BillieMantzOik/VICTRA/data.

*Benefit‐Sharing Statement*: A research collaboration was developed with scientists from Panama and Costa Rica, where genetic samples of studied species were collected. All collaborators are included as co‐authors. This research adheres to the principles of open science, ensuring that all data, scripts, and methodologies are freely accessible to the scientific community. We encourage the use of these resources to facilitate reproducibility, further research, and collaborative advancements in the field.

## Conflicts of Interest

The authors declare no conflicts of interest.

## Supporting information


**Data S1:** mec70214‐sup‐0001‐Supinfo.zip.

## Data Availability

The data that support the findings of this study are openly available in zenodo at https://doi.org/10.5281/zenodo.17714586, reference number 10.5281/zenodo.17714587.
