# Supplementary material for: Pigments, Chromatophore Structure, and Gene Expression Underlying Colour Polytypy of a Panamanian Poison Frog
Source: Mol Ecol. 2025 Dec 22;35(1):e70214. doi: 10.1111/mec.70214 (PMC12745852; doi:10.1111/mec.70214)
Supplement: Supplementary file 1 — Data S1: mec70214‐sup‐0001‐Supinfo.zip. [file MEC-35-e70214-s001.zip › mec70214-sup-0001-SupMat1.docx]

**Supplementary Information SM2:**

**Section SM2. 1:**

Chromatographic Conditions:

Chromatographic separation of carotenoids was performed using an ACQUITY™ Premier BEH C18 column (1.7 µm, 2.1 × 100 mm) with an injection volume of 1 µL. The mobile phase consisted of:

Solvent A: Methanol:acetonitrile (3:7, v/v)

Solvent B: Water with 0.01% (v/v) formic acid

The flow rate was maintained at 0.4 mL/min throughout the gradient. The elution program for oxygenated carotenoids was as follows:

| Time (min) | % Solvent A | % Solvent B | Gradient Description |
| --- | --- | --- | --- |
| 0.0–0.5 | 60% | 40% | Initial conditions |
| 0.5–3.0 | Linear ramp |  | Increase Solvent A to 70% |
| 3.0–4.0 | Linear ramp |  | Increase Solvent A to 90% |
| 4.0–7.0 | 90% | 10% | Isocratic hold |
| 7.0–9.0 | — | — | Re-equilibration to initial conditions |
| 9.0–10.0 | 60% | 40% | Return to starting conditions |

These conditions enabled the efficient separation of target analytes within a 10-minute total runtime.

For beta-carotene analysis the following elution program was follow.

| Time (min) | % Solvent A | | % Solvent B | | Gradient Description | |  |
| --- | --- | --- | --- | --- | --- | --- | --- |
| 0.0–0.5 | | 60% | | 40% | | Initial conditions | |
| 0.5–3.0 | | Linear ramp | |  | | Increase Solvent A to 70% | |
| 3.0–4.0 | | Linear ramp | |  | | Increase Solvent Ato 90% | |
| 4.0–11.0 | | 90% | | 10% | | Isocratic hold | |
| 11-12 | | Linear ramp | |  | | Increase Solvent A to 100% | |
| 12-22.9 | | 100 | | 10 | | Isocratic Hold | |
| 22.9-23.0 | | --- | | --- | | Re-equilibration to initial conditions | |
| 23-25 | | 60 | | 40 | | Return to starting conditions | |

Calibration Curve

Quantification was performed by constructing calibration curves using two reference standards:

Astaxanthin (Sigma-Aldrich) was used to quantify oxygenated carotenoids. The standard curve was generated with concentrations of 11.19, 8.95, 6.71, 4.47, 2.24, and 1.11 ng/mL, yielding a correlation coefficient (R²) of 0.9943. Quantification was based on the extracted ion chromatogram (EIC) for the m/z range 597.38–597.39 in positive ESI mode. Carotenoids were quantified relative to Astaxanthin, assuming similar ionization efficiency under the applied conditions.

β-Carotene (Sigma-Aldrich) was used for non-oxygenated carotenoids. A calibration curve was built using concentrations of 2.38, 11.90, 47.84, 54.63, 60.33, 62.64, and 118.28 ng/mL, with an R² of 0.996. The EIC was extracted in the m/z range 537.44–537.45 using positive APCI mode. Quantification was again performed relative to the standard, assuming comparable ionization efficiency.

Mass Spectrometric Detection

Mass spectrometry was performed using a Orbitrap Exploris 120 (Thermo Scientific) high resolution spectrometer, in positive ion mode using both data-dependent acquisition (DDA) and Full MS scan modes. The H-ESI source was operated with a spray voltage of 3400 V, sheath gas at 60 arb, auxiliary gas at 15 arb, and sweep gas at 2 arb. The ion transfer tube and vaporizer temperatures were set to 350 °C.

Full MS scans were acquired at a resolution of 60,000 FWHM over an m/z range of 100–1500, with the RF lens set to 60%. The AGC target was set to “Standard”, the maximum injection time to “Auto”, and data were collected in centroid mode with one microscan per scan.

MS/MS spectra were triggered based on precursor ion intensity thresholds using higher-energy collisional dissociation (HCD). Dynamic exclusion and isotope exclusion were enabled to improve scan efficiency. Each full MS scan was followed by up to three MS² scans per cycle, triggered upon apex detection.

For MS/MS acquisition, the following parameters were applied:

- Isolation window: 0.5 m/z

- Isolation offset: 0 m/z

- HCD collision energy: 30% and 50% a

- Resolution: 15,000 (FWHM)

- Scan range mode: Auto

- AGC target: 50% (custom normalized)

- Maximum injection time: 125 ms (custom mode)

- Microscans: 1

When operated in APCI mode, the source was set with a static spray current, sheath gas at 10 arb, auxiliary gas at 5 arb, and sweep gas at 2 arb. Other acquisition parameters remained as described above.

Data processing

Raw spectra were processed with Compound Discoverer version 3.3 SP3 (Thermo Fisher Scientific, USA). An untargeted metabolomics workflow was applied with a mass tolerance of 5 ppm, a signal-to-noise ratio threshold of 1.5, a minimum peak intensity of 10,000, and a minimum of five scans per peak. Feature extraction included peak detection, chromatographic alignment, and background subtraction using blank samples. Chromatographic features were aligned across samples based on retention time using an adaptive curve model with a tolerance of ±0.2 minutes, and a feature table was generated listing all chromatographic peaks contributing to each compound. Compounds selected for annotation following MS2 acquisition were processed using integrated spectral matching workflows within Compound Discoverer 3.3 SP3, including mzCloud Search for direct spectral matches, which were assigned a mzCloud Score and Confidence Level, and mzLogic Search for indirect matches, which combined spectral similarity with ChemSpider structural databases to generate a mzLogic Score. In silico fragmentation prediction was performed using Compound Discoverer 3.3 SP3 and the HighChem Fragmentation Library, with resulting annotations evaluated using the FISh scoring algorithm to assess spectral match quality. For each compound, the proposed structure with the highest combined mzCloud Score, mzLogic Score, and FISh Score were selected for downstream analysis.

UV absorption spectra (180–900 nm) were extracted for each annotated compound when feasible, and spectral profiles were compared against published reference spectra for known carotenoids (e.g., β-carotene, asthaxanthin, canthaxantin) to support compound identification.

Carotenoid quantitation was conducted using Thermo Xcalibur version 4.5. Chromatographic peaks corresponding to annotated carotenoids were detected and integrated using the ICIS (Integrated Component Identification System) algorithm

Except for Astaxanthin and b-carotene, the rest of the carotenoids are annotated with level 2 (Shymansky et al.) and should be considered as putative suggestions

**Table SM2.1.** Characteristics and molecular weights used in the annotation of the compounds detected during the screening of *Oophaga vicentei* pterins.

| Structure | Compound | Molecular Formula | Precursor (m/z) |
| --- | --- | --- | --- |
| 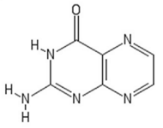 | Pterin | C_6_H_5_N_5_O | [M + H]^+^ : 164.0567^2^ |
| 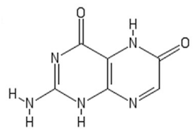 | Xanthopterin^4,5^ | C_6_H_5_N_5_O_2_ | [M + H]^+^ : 180.0516^2^ |
| 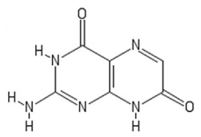 | Isoxanthopterin^4,5^ | C_6_H_5_N_5_O_2_ | [M + H]^+^ : 180.0516^2^ |
| 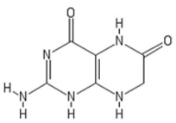 | 7,8-dihydroxanthopterin | C_6_H_7_N_5_O_2_ | [M + H]^+^ : 182.0672^2^ |
| 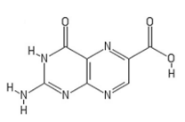 | 6-carboxipterina^4^ | C_7_H_5_N_5_O_3_ | [M + H]^+^ : 208.0462^2^ |
| 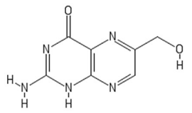 | 6-hydroximetilpterina | C_7_H_7_N_5_O_2_ | [M + H]^+^ : 194.0672^2^ |
| 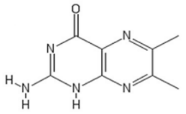 | 6,7-dimethylpterin | C_8_H_9_N_5_O | 189.874  [M + H]^+^ : 192.0880^2^ |
| 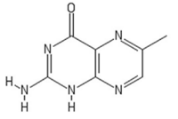 | 6-metilpterina | C_7_H_7_N_5_O | [M + H]^+^: 178.0723^2^ |
| 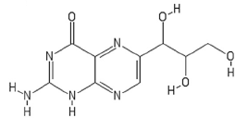 | 6-Neopterina | C_9_H_11_N_5_O_4_ | 251.987^1^  [M + H]^+^ : 254.0884^2^  254.0/206.2 (ion pair)^3^  254.0/236.3 (ion pair) |
|  | 7-neopterina | C_9_H_11_N_5_O_4_ | [M + H]^+^ : 254.0889 |
| 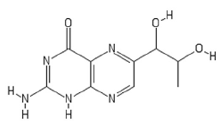 | 6-biopterina^4^ | C_9_H_11_N_5_O_3_ | [M + H]^+^ : 238.0935^1^ |
|  | 7-biopterina | C_9_H_11_N_5_O_3_ | [M + H]^+^ : 238.0935 |
| 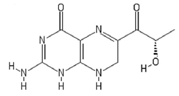 | Sepiapterin^5,6^ | C_9_H_11_N_5_O_3_ | [M + H]^+^ : 238.0935^2^  238.1/192.3 (ion pair)^3^  238.1/165.3 |
| 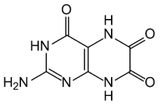 | Leucopterin^5^ | C_6_H_5_N_5_O_3_ | [M + H]^+^ : 196.0471 |
| 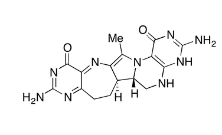 | Drosopterin^5^ | C_16_H_17_N_9_O_2_ | [M + H]^+^ : 368.1583  369.14^7^  product ions (m/z): 152.1, 230.2, and 353.2^7^ |
| 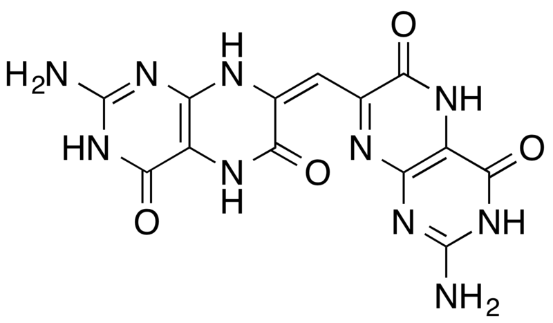 | Pterorodin^5^  (Rodopterin) | C_13_H_10_N_10_O_4_ | [M + H]^+^ : 371.0965 |

**References**

Allegri, G., Costa Netto, H. J. B., Ferreira Gomes, L. N. L., Costa De Oliveira, M. L., Scalco, F. B., & De Aquino Neto, F. R. (2012). Determination of six pterins in urine by LC–MS/MS. *Bioanalysis, 4*(14), 1739–1746. doi: 10.4155/bio.12.131

Burton, C., Weng, R., Yang, L., Bai, Y., Liu, H., & Ma, Y. (2015). High-throughput intracellular pteridinic profiling by liquid chromatography–quadrupole time-of-flight mass spectrometry. *Analytica Chimica Acta, 853*, 442–450. doi: 10.1016/j.aca.2014.10.044

Burton, C., Shi, H., & Ma, Y. (2016). Development of a high-performance liquid chromatography–tandem mass spectrometry urinary pterinomics workflow. *Analytica Chimica Acta, 927*, 72–81. doi: 10.1016/j.aca.2016.05.005

Suga, T., & Munesada, K. (1988). The pigments in the dorsal skin of frogs. *Journal of Natural Products, 51*(4), 713–718. doi: 10.1021/np50058a008

Andrade, P., & Carneiro, M. (2021). Pterin-based pigmentation in animals. *Biology Letters, 17*(8), 20210221. doi: 10.1098/rsbl.2021.0221

Hama, T., & Obika, M. (1960). Pterin synthesis in the amphibian neural crest cell. *Nature, 187*(4734), 326–327. doi: 10.1038/187326a0

Phan, N. T., Munem, M., Ewing, A. G., & Fletcher, J. S. (2017). MS/MS analysis and imaging of lipids across Drosophila brain using secondary ion mass spectrometry. *Analytical and Bioanalytical Chemistry, 409*(16), 3923–3932. doi: 10.1007/s00216-017-0336-4

Russell, P. B., Purrmann, R., Schmitt, W., & Hitchings, G. H. (1949). The synthesis of pterorhodin (rhodopterin). *Journal of the American Chemical Society, 71*(10), 3412–3416. doi: 10.1021/ja01178a042

Schymanski, E. L., et al. (2014). Identifying small molecules via high resolution mass spectrometry: Communication confidence. *Environmental Science & Technology, 48*(4), 2097–2098. doi: 10.1021/es5002105
