# Supplementary material for: Pigments, Chromatophore Structure, and Gene Expression Underlying Colour Polytypy of a Panamanian Poison Frog
Source: Mol Ecol. 2025 Dec 22;35(1):e70214. doi: 10.1111/mec.70214 (PMC12745852; doi:10.1111/mec.70214)
Supplement: Supplementary file 1 — Data S1: mec70214‐sup‐0001‐Supinfo.zip. [file MEC-35-e70214-s001.zip › mec70214-sup-0002-SupMat2.docx]

**Supplementary Material 1**

**Figures**

**Figure A1.:** Average reflectance spectra for each of the four morphs of *Oophaga vicentei*: aquamarine (aquamarine line), brown (brown line), green (green line), and red (red line). Each curve represents the mean reflectance across individuals of that morph.

xanthophylls

Concentration ng/g

**Figure A2.:** Boxplots showing the estimated measures per sample, grouped by morph for histological variables (A) and pigment concentrations (B). **A.** Boxplots show measurements in nm of chromatophore layer thickness (iridophore, melanophore, xanthophore) grouped by morph. **B.** Boxplots show concentrations per pigment class type (ketocarotenoids, xanthophylls, xanthopterin) grouped by morph and per sample in ng/g.


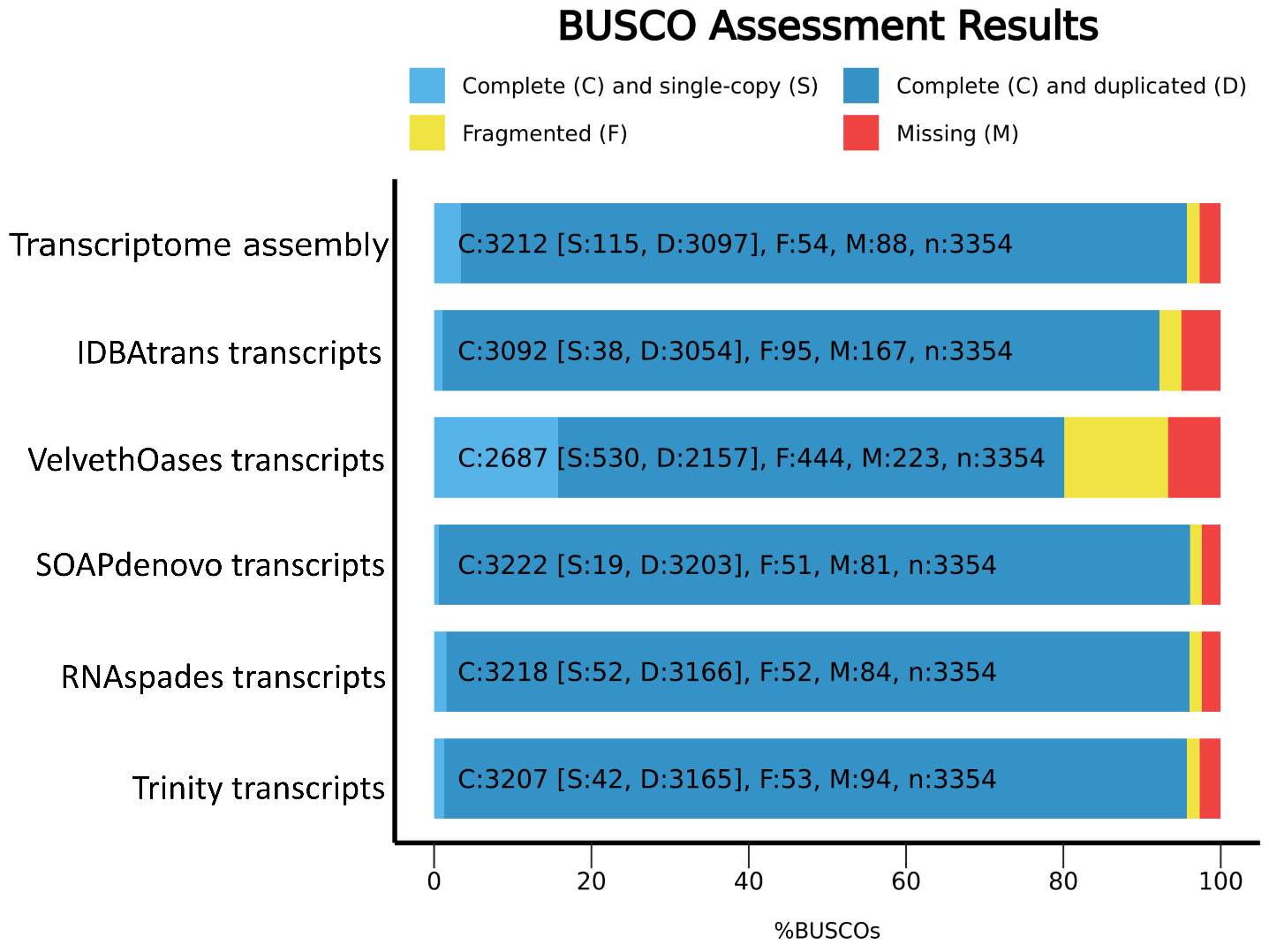

**Figure A3.:** BUSCO assessment of transcriptome assemblies for each assembler and the EviGene Consensus transcriptome. The bar plot displays the completeness of each transcriptome assembly based on Benchmarking Universal Single-Copy Orthologs (BUSCO). Each bar represents a different assembly, categorized by completeness metrics: Complete (C) genes (both single-copy and duplicated, shown in blue), Fragmented (F) genes (yellow), and missing (M) genes (red). Color indicates completeness metrics. Results are shown for different assemblers (IDBATrans transcripts, VelvetOases transcripts, SOAPdenovo transcripts, rnaSPADES transcripts and Trinity transcripts), providing a comparative assessment of their performance and for the Transcript collection.

EviGene Consensus transcriptome

IDBA trans

VelvethOases

SOAPdenovo


RNAspades

Trinity


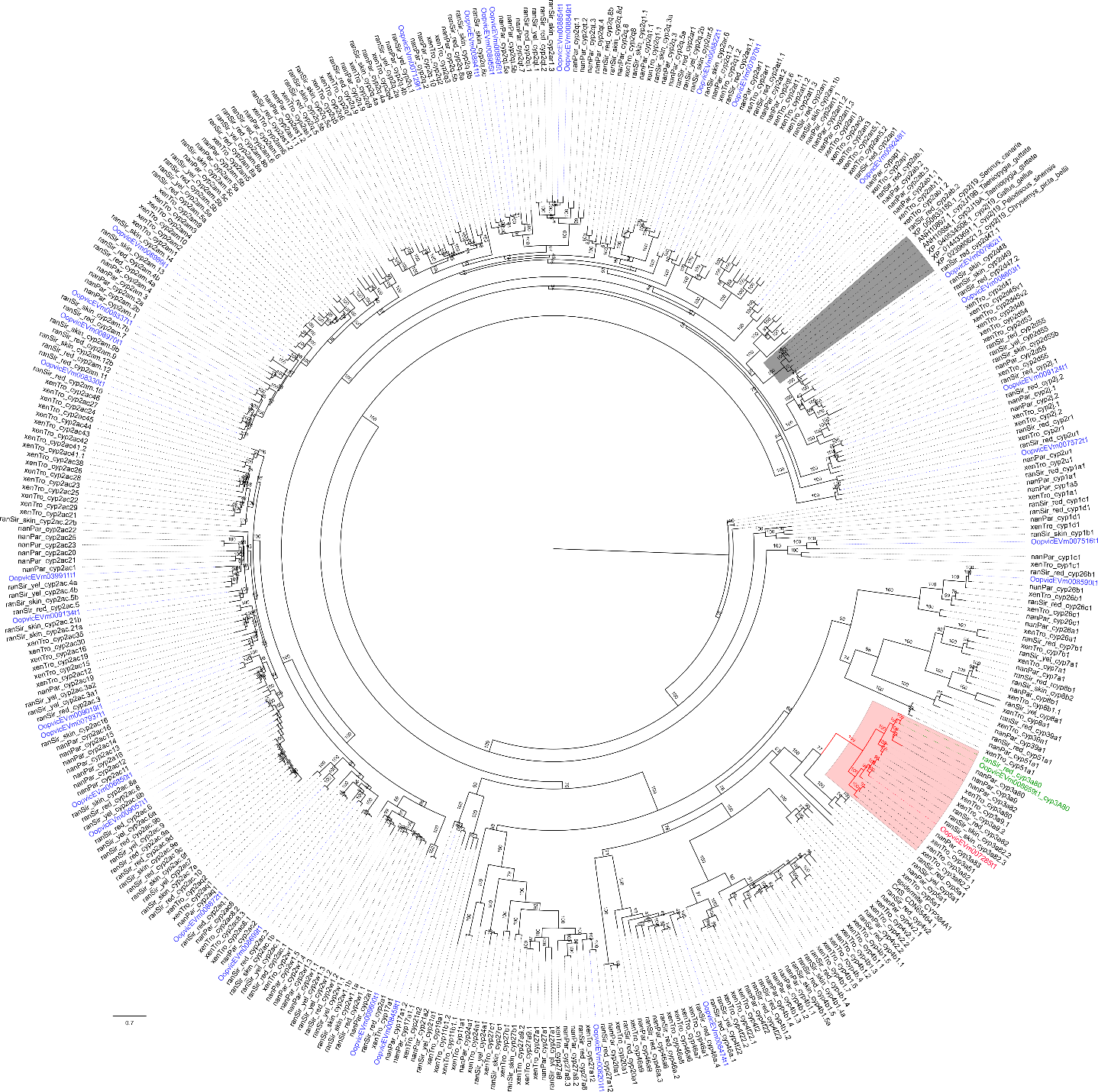


**Figure A4.:** Phylogenetic relationships between anuran cytochrome p450 sequences and homologous sequences with differential expression between *Oophaga vicentei* color phenotypes. The maximum likelihood inference tree was obtained using a JTT+F+R8 protein substitution model (best fit according to its BIC) with node support evaluated with 1000 ultrafast bootstrap pseudo-replicates. Differentially expressed sequences of *O. vicentei* are highlighted in blue. The clades containing known ketolase candidates of birds (*cyp2j19* clade) and poison frogs (*cyp3A* clade) are shaded in gray and red, respectively. The candidate ketolase of *O. vicentei* (*cyp3A82*, highlighted in red) belongs to the cyp3A clade but differs from the *Ranitomeya sirensis* ketolase and its *O. vicentei* ortholog (*cyp3A80*, green) which shows no differential expression.

**
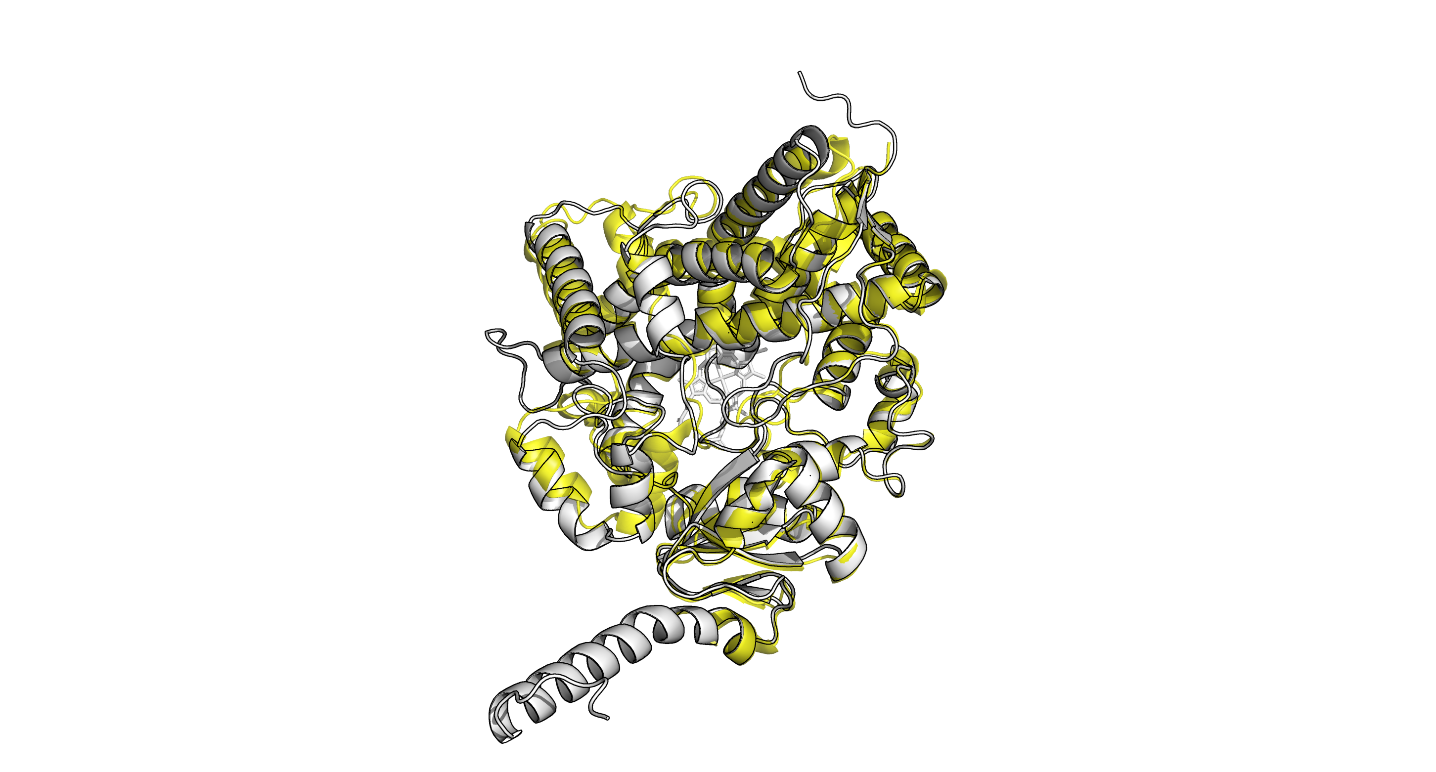
**

**Figure A5.:** Structural alignment of the homology model of *O. vicentei* ketolase (grey cartoon) and human cytochrome P450 3A4 (CYP3A4) (yellow cartoon).

**
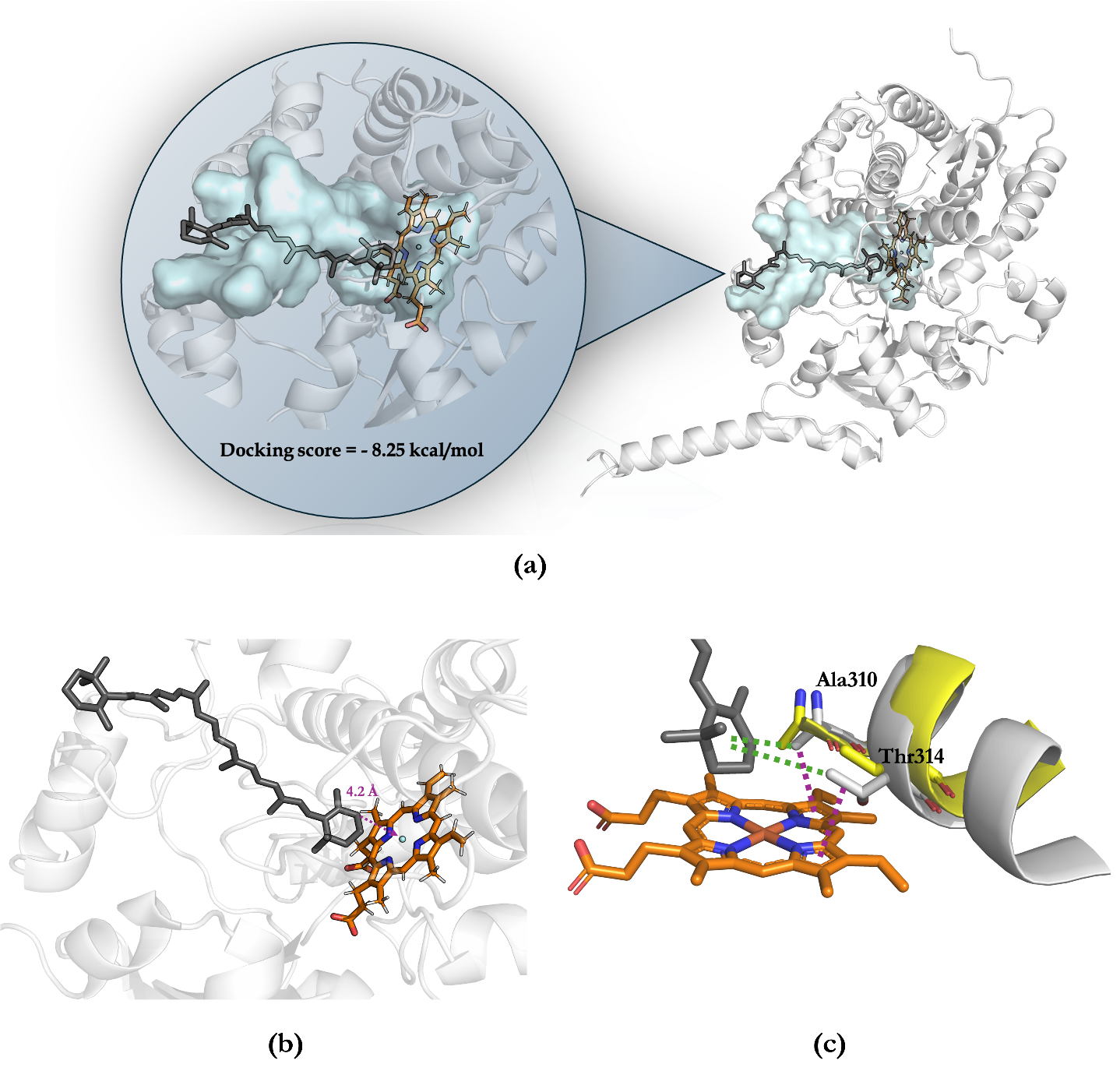
**

**Figure A6.: (a)** The predicted β-carotene (black sticks) pose in the ketolase (grey cartoon) discovered from *Oophaga vicentei.* The binding site (light blue color surface) at the ketolase includes the heme prosthetic group (C, N, O, and Fe atoms in orange, blue, red, and light blue, respectively). **(b)** The β-ionone C4 atom in β-carotene resides over the heme prosthetic group to ~4.2 Å (purple arrow) which positions it adequately for subsequent C4 ketolation. **(c)** Residues Ala310 and Thr314 from the conserved region of *O. vicentei* ketolase (grey cartoon) form hydrophobic interactions with both the β-ionone ring (black sticks) and the heme prosthetic group, indicated by green and purple dashed lines, respectively. For comparison, the corresponding residues Ala305 and Thr309 from the conserved region of human cytochrome P450 3A4 (CYP3A4) are also shown (yellow cartoon).


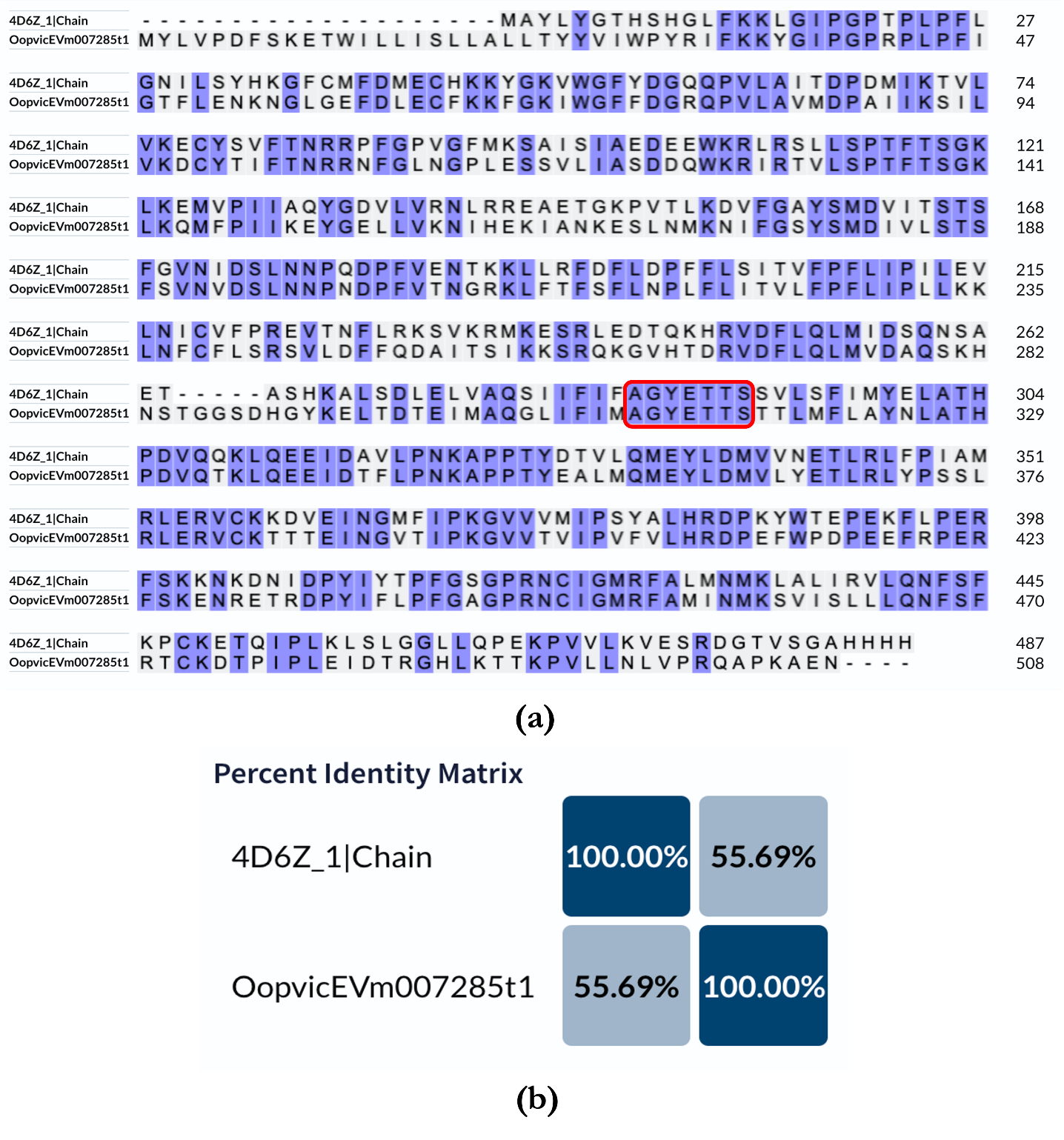


**Figure A7.: (a)** Sequences alignment of the human cytochrome P450 3A4 (4D6Z_1|Chain) and the *O. vicentei* ketolase (OopvicEVm007285t1). Amino acids marked in purple represent conserved regions in both sequences. The region enclosed in red represents a conserved region of the binding site of both proteins. (**b)** Percent identity matrix for the sequence of *O. vicentei* ketolase and human cytochrome P450 3A4 (CYP3A4).

**Supplementary Material 1**

**Tables**

**Table A1.:** Transcriptome assembly metrics under different reference genome conditions for Oophaga vicentei. The table compares de novo and genome-guided assembly approaches using RNA-seq data. Columns indicate: (i) Reference genome used for assembly (or none for de novo), (ii) Read alignment rate (mean percentage of reads mapped), (iii) Assembly method, (iv) Number of transcripts assembled, (v) N50 contiguity metric (in base pairs), and (vi) Vertebrata BUSCO score (%) representing assembly completeness. Higher BUSCO scores and N50 values indicate better assembly quality.

| **reference genome** | **read alignment rate (mean, in %)** | **assembly method** | **nr. of transcripts** | **N50** | **vertebrata BUSCO score (%)** |
| --- | --- | --- | --- | --- | --- |
| none | 87.1 | de novo (EviGene) | 836,454 | 1862 | 95.8 |
| *O. pumilio* [GCA_009801035.1] | 68.2 | genome-guided (Trinity) | 463,883 | 927 | 62.1 |
| *O. sylvatica* [GCA_033576555.1] | 55.0 | genome-guided (Trinity) | 599,391 | 624 | 54.2 |

**Table A2.:** Transcriptome Assembly Metrics Across Different Assemblers. This table presents key assembly metrics for transcriptomes generated using five different de novo transcriptome assemblers: IDBA-Tran, OASES, SOAPdenovo-Trans, rnaSPADES, and Trinity. The "Collection" column represents the combined dataset from all assemblers and the EviGene consensus transcriptome refers to the final reference transcriptome, used in downstream analysis, after the evigene pipeline. Metrics include the total number of assembled transcripts, the number of transcripts exceeding 500 bp and 1000 bp, average transcript length, the longest assembled transcript, total assembly length, and N50 values. N50 represents the length at which 50% of the total assembled bases are contained in transcripts of at least this length, serving as a measure of assembly contiguity.

| Metrics | Idba_trans | OASES | SOAP_denovo | rnaSPADES | Trinity | EviGene Consensus transcriptome |
| --- | --- | --- | --- | --- | --- | --- |
| Transcripts | 1342755 | 2686105 | 3448181 | 1165592 | 1912755 | 836,454 |
| Transcripts > 500bp | 882595 | 1012314 | 979384 | 359330 | 517890 | 432,400 |
| Transcripts > 1000bp | 578833 | 336121 | 522682 | 205833 | 294035 | 239,562 |
| Average transcripts length | 1523.689 | 564.052 | 666.031 | 790.331 | 657.672 | 1100.6 |
| Longest transcript | 45952 | 23539 | 49088 | 49714 | 34429 | 369,61 |
| Total length | 2045941694 | 1515103669 | 2296596598 | 921202992 | 1257966000 | 752,746,335 |
| Transcript N50 | 2849 | 712 | 1394 | 1840 | 1354 | 1862 |

References

Andersson, S. (1999). Morphology of UV reflectance in a whistling-thrush: Implications for the study of structural colour signalling in birds. *Journal of Avian Biology, 30*(2), 193–204. doi: 10.2307/3677123

Andersson, S., Ornborg, J., & Andersson, M. (1998). Ultraviolet sexual dimorphism and assortative mating in blue tits. *Proceedings of the Royal Society B: Biological Sciences, 265*(1395), 445–450. doi: 10.1098/rspb.1998.0315

Andersson, S., Pryke, S., Ornborg, J., Lawes, M., & Andersson, M. (2002). Multiple receivers, multiple ornaments, and a trade-off between agonistic and epigamic signaling in a widowbird. *The American Naturalist, 160*(5), 683–691. doi: 10.1086/342817

Chan, I. Z. W., Chang, J. J. M., Huang, D., & Todd, P. A. (2019). Colour pattern measurements successfully differentiate two cryptic Onchidiidae Rafinesque, 1815 species. *Marine Biodiversity, 49*(4), 1743–1750. doi: 10.1007/s12526-018-0936-7

Delhey, K., Johnsen, A., Peters, A., Andersson, S., & Kempenaers, B. (2003). Paternity analysis reveals opposing selection pressures on crown coloration in the blue tit (*Parus caeruleus*). *Proceedings of the Royal Society B: Biological Sciences, 270*(1528), 2057–2063. doi: 10.1098/rspb.2003.2471

Keyser, A., & Hill, G. (1999). Condition-dependent variation in the blue-ultraviolet coloration of a structurally based plumage ornament. *Proceedings of the Royal Society B: Biological Sciences, 266*(1424), 771–777. doi: 10.1098/rspb.1999.0704

Keyser, A. J., & Hill, G. (2000). Structurally based plumage coloration is an honest signal of quality in male blue grosbeaks. *Behavioral Ecology, 11*(2), 202–209. doi: 10.1093/beheco/11.2.202

Ligon, R. A., Diaz, C. D., Morano, J. L., Troscianko, J., Stevens, M., Moskeland, A., … Scholes, E. (2018). Evolution of correlated complexity in the radically different courtship signals of birds-of-paradise. *PLOS Biology, 16*(11), e2006962. doi: 10.1371/journal.pbio.2006962

Maia, R., Eliason, C. M., Bitton, P. P., Doucet, S. M., & Shawkey, M. D. (2013). pavo: An R package for the analysis, visualization and organization of spectral data. *Methods in Ecology and Evolution, 4*(10), 906–913. doi: 10.1111/2041-210X.12069

Ornborg, J., Andersson, S., Griffith, S., & Sheldon, B. (2002). Seasonal changes in a ultraviolet structural colour signal in blue tits (*Parus caeruleus*). *Biological Journal of the Linnean Society, 76*(2), 237–245. doi: 10.1046/j.1095-8312.2002.00061.x

Peters, A., Denk, A., Delhey, K., & Kempenaers, B. (2004). Carotenoid-based bill colour as an indicator of immunocompetence and sperm performance in male mallards. *Journal of Evolutionary Biology, 17*(6), 1111–1120. doi: 10.1111/j.1420-9101.2004.00743.x

Pryke, S., Lawes, M., & Andersson, S. (2001). Agonistic carotenoid signalling in male red-collared widowbirds: Aggression related to the colour signal of both the territory owner and model intruder. *Animal Behaviour, 62*(4), 695–704. doi: 10.1006/anbe.2001.1807

Saks, L., McGraw, K., & Horak, P. (2003). How feather colour reflects its carotenoid content. *Functional Ecology, 17*(4), 555–561. doi: 10.1046/j.1365-2435.2003.00765.x

Shawkey, M., Estes, A., Siefferman, L., & Hill, G. (2003). Nanostructure predicts intraspecific variation in ultraviolet-blue plumage colour. *Proceedings of the Royal Society B: Biological Sciences, 270*(1523), 1455–1460. doi: 10.1098/rspb.2003.2390

Siefferman, L., & Hill, G. (2005). UV-blue structural coloration and competition for nestboxes in male eastern bluebirds. *Animal Behaviour, 69*(1), 67–72. doi: 10.1016/j.anbehav.2004.05.008

Smiseth, P., Ornborg, J., Andersson, S., & Amundsen, T. (2001). Is male plumage reflectance correlated with paternal care in bluethroats? *Behavioral Ecology, 12*(2), 164–170. doi: 10.1093/beheco/12.2.164

Winters, A. E., Wilson, N. G., van den Berg, C. P., How, M. J., Endler, J. A., Marshall, N. J., … Cheney, K. L. (2018). Toxicity and taste: Unequal chemical defences in a mimicry ring. *Proceedings of the Royal Society B: Biological Sciences, 285*(1880), 20180457. doi: 10.1098/rspb.2018.0457
